# A Structural Basis for BRD2/4-Mediated Host Chromatin Interaction and Oligomer Assembly of Kaposi Sarcoma-Associated Herpesvirus and Murine Gammaherpesvirus LANA Proteins

**DOI:** 10.1371/journal.ppat.1003640

**Published:** 2013-10-17

**Authors:** Jan Hellert, Magdalena Weidner-Glunde, Joern Krausze, Ulrike Richter, Heiko Adler, Roman Fedorov, Marcel Pietrek, Jessica Rückert, Christiane Ritter, Thomas F. Schulz, Thorsten Lührs

**Affiliations:** 1 Department of Structural Biology, Helmholtz Centre for Infection Research, Braunschweig, Germany; 2 Institute of Virology, Hannover Medical School, Hannover, Germany; 3 Research Unit Gene Vectors, Helmholtz Zentrum München, German Research Center for Environmental Health (GmbH), Munich, Germany; 4 Research Division for Structural Analysis, Hannover Medical School, Hannover, Germany; 5 Institute of Biophysical Chemistry, Hannover Medical School, Hannover, Germany; University of Southern California Keck School of Medicine, United States of America

## Abstract

Kaposi sarcoma-associated herpesvirus (KSHV) establishes a lifelong latent infection and causes several malignancies in humans. Murine herpesvirus 68 (MHV-68) is a related γ_2_-herpesvirus frequently used as a model to study the biology of γ-herpesviruses *in vivo*. The KSHV latency-associated nuclear antigen (kLANA) and the MHV68 mLANA (orf73) protein are required for latent viral replication and persistence. Latent episomal KSHV genomes and kLANA form nuclear microdomains, termed ‘LANA speckles’, which also contain cellular chromatin proteins, including BRD2 and BRD4, members of the BRD/BET family of chromatin modulators. We solved the X-ray crystal structure of the C-terminal DNA binding domains (CTD) of kLANA and MHV-68 mLANA. While these structures share the overall fold with the EBNA1 protein of Epstein-Barr virus, they differ substantially in their surface characteristics. Opposite to the DNA binding site, both kLANA and mLANA CTD contain a characteristic lysine-rich positively charged surface patch, which appears to be a unique feature of γ_2_-herpesviral LANA proteins. Importantly, kLANA and mLANA CTD dimers undergo higher order oligomerization. Using NMR spectroscopy we identified a specific binding site for the ET domains of BRD2/4 on kLANA. Functional studies employing multiple kLANA mutants indicate that the oligomerization of native kLANA CTD dimers, the characteristic basic patch and the ET binding site on the kLANA surface are required for the formation of kLANA ‘nuclear speckles’ and latent replication. Similarly, the basic patch on mLANA contributes to the establishment of MHV-68 latency in spleen cells *in vivo*. In summary, our data provide a structural basis for the formation of higher order LANA oligomers, which is required for nuclear speckle formation, latent replication and viral persistence.

## Author Summary

Kaposi sarcoma-associated herpesvirus (KSHV) causes Kaposi Sarcoma, Primary Effusion lymphoma and the plasma cell variant of Multicentric Castleman's Disease. Its oncogenic effect is linked to its ability to persist in a latent form for the life time of infected individuals. During latency viral genomes are replicated and passed to daughter cells in synchrony with the infected cell without the formation of new virions. A key viral protein in this process is the latency-associated nuclear antigen, LANA. In latently infected cells, viral genomes and LANA form characteristic nuclear microdomains, termed ‘LANA speckles’, which also contain cellular chromatin components. We have solved the crystal structure of the c-terminal, DNA-binding, domain (CTD) of KSHV LANA (kLANA) and its homologue mLANA of a related murine γ_2_-herpesvirus, which is frequently used as a model to study latent persistence *in vivo*. We also identified the binding site for two chromatin proteins, BRD2/4, by NMR spectroscopy. We demonstrate the functional importance of these structural features, and their contribution to latent replication and ‘LANA speckle’ formation, in cell culture and *in vivo* experiments. Our results provide a structural basis for the assembly of LANA-containing nuclear structures that are required for latent viral replication and persistence.

## Introduction

Kaposi sarcoma-associated herpesvirus (KSHV, also HHV8), the cause of Kaposi Sarcoma and two lymphoid neoplasms, can persist in a latent form in infected endothelial and B-cells [1]. The key player in the regulation of KSHV latency is the latency-associated nuclear antigen (LANA), which is detected in all KSHV infected cells [2], [3] and is required for the latent episomal replication of this virus [4], [5]. The terminal repeat (TR) region of KSHV contains 20–40 TR elements, which constitute the latent origin of replication [5], [6]. The C-terminal domain (CTD) of LANA forms dimers, which bind two adjacent DNA sequences, the LANA binding sites (LBS1 & LBS2) in a single TR [4], [7]. Thus, with respect to DNA binding, LANA resembles other viral origin binding proteins including EBNA-1 of Epstein-Barr virus (EBV) [8] and E2 of human papillomavirus (HPV) [9], [10]. LANA also recruits components of the cellular replication machinery to the KSHV latent origin of replication, thereby allowing the virus to replicate its latent episome in the S phase of the cell cycle along with the cellular chromatin [11]. It also regulates transcription of both viral [12], [13] and cellular genes [14], [15], [16], [17], [18].

In latently infected cells LANA and the viral episomes are concentrated in characteristic nuclear speckles [3], [19], [20], [21]. To ensure the partitioning of newly synthesized genomes to daughter cells during mitosis LANA tethers KSHV genomes to host mitotic chromosomes by attaching its N-terminal domain to histones H2A/B [22]. In addition, the LANA CTD also binds to mitotic chromosomes and interacts with chromatin-associated proteins [22], [23], [24], [25] including members of the BET (Bromodomain and ET domain) family [26], [27], [28]. Increasing evidence points to an important role of BET family members in the life cycle of several DNA tumor virus families. BRD4 has been shown to be involved in the tethering of bovine papillomavirus E2 protein and associated episomal viral DNA to host mitotic chromosomes [29]. Moreover the human papillomavirus (HPV) E2 proteins act as transcriptional regulators, and this function also involves BRD4 [30], [31], [32]. In addition, BRD4 has also been implicated in the latent replication of Merkel cell polyoma virus [33]. For LANA, there is evidence that BRD4 contributes to the recruitment of this protein to chromatin and to its transcriptional properties [26], [28] while BRD2 may also affect the role of LANA as a transcriptional activator and is able to phosphorylate the LANA CTD [27]. Thus, BET proteins play a pivotal role in the life cycles of multiple dsDNA viruses that cause latent infections in the host.

Murine herpesvirus 68 (MHV-68) is a γ2-herpesvirus, which provides an *in vivo* model for the study of KSHV [34], [35]. The open reading frame 73 (orf73) of MHV-68 encodes mLANA, which is distantly related to KSHV LANA (in the following referred to as kLANA). Similarly to kLANA, mLANA binds to specific sites within the TR region of the MHV-68 genome, is required for the latent replication and for establishment of latency in splenic B-cells *in vivo* [36], [37], [38], [39]. The ability of mLANA to act as a transcriptional activator may be linked to its recruitment of BRD2 or BRD4 [40].

Here we report the crystal structures of both kLANA and mLANA CTDs. Both structures display unexpected features. We show that LANA dimers form oligomers and that chromatin associated BET proteins bind two sites on kLANA. Both LANA oligomerization and BET binding contribute to latent replication, nuclear speckle formation and persistence of γ2-herpesvirses, *in vivo* and *in vitro*.

## Results

### The 3D Structures of KSHV and MHV-68 LANA C-Terminal Domains

**Figure 1:**
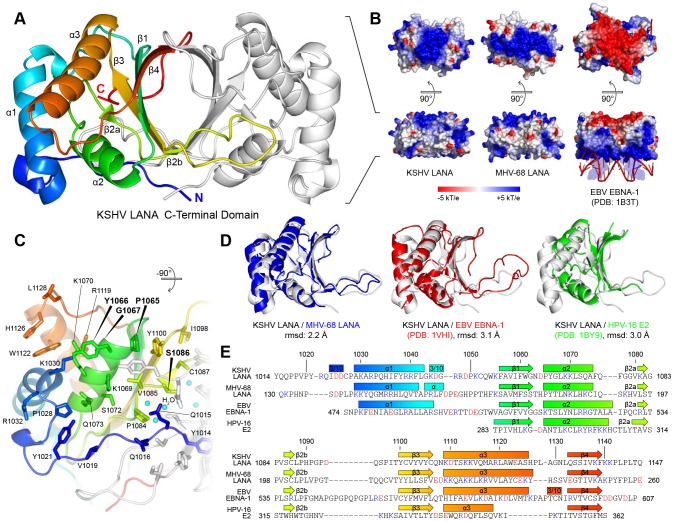
Crystal Structure of the KSHV LANA CTD with Its Orthologs. **A**: Crystal structure of the dimeric kLANA CTD, front view. **B**: Surface electrostatic potential of kLANA, mLANA, and the EBV EBNA-1 CTD in front view (bottom) and top view (above). Red represents negative charge and blue represents positive charge. **C**: Residues at the sequence specific DNA binding site of kLANA, bottom view. Residues mutated in this study are bold faced. **D**: 3D superpositions of the kLANA CTD (white) with mLANA (blue), EBV EBNA-1 (red), and HPV-16 E2 (green) CTD monomers. **E**: Structure-based sequence alignment of kLANA, mLANA, EBV EBNA-1, and HPV-16 E2 CTDs. Acidic residues are red and basic residues are blue. See also Figure S1.

The structure of the kLANA fragment comprising residues 1013–1149 was solved by X-ray crystallography at a resolution of 2.60 Å (Figure 1A,B), where electron density was observed for residues 1014–1147. kLANA(1013–1149) forms dimers that are stabilized by an eight-stranded antiparallel intermolecular β-barrel to which each monomer contributes four β-strands (β1–β4). In addition to the stabilization by the hydrogen bonding network of the β-barrel, hydrophobic amino acid side chains project into the core and form a tightly packed hydrophobic cluster. The total dimer interface area is 2080 Å^2^ indicating that LANA is an obligate dimer [41]. Strand β2 contains a β-bulge, which lies directly above a cluster of six water molecules that are observed in the electron density map (Figure S1A). These form a hydrogen bonding network with the amino acid side chains Y1103 and Y1105 that are also part of the dimer interface. The β-barrel is flanked by two α helices, α2 and α3. The N-terminal helix, α1, is packed against the two other helices and is not in direct contact with the central β-barrel.

We also solved the crystal structure of a C-terminal fragment of MHV-68 LANA, mLANA(124–260), at a resolution of 2.14 Å, where a detailed structure was obtained for residues 130–260 (Figures S1B,C). It also is a native dimer, and the fold is very well conserved with kLANA (Figure 1D). Moreover, the two LANA structures share the same fold with the 3D structures of the dimeric DNA binding domains of Epstein-Barr virus' EBNA-1 [42] and papillomavirus E2 [43], [44] (Figures 1D,E), even though the corresponding sequence identities are only 16% and 10%, respectively (Figures S1D,E). However, the structured CTDs of kLANA and mLANA share 28% sequence identity and the root-mean square deviation (r.m.s.d.) of the peptide backbone is only 2.2 Å. The r.m.s.d. between the CTDs of kLANA and EBNA-1 of 3.1 Å is significantly larger (Figure 1D). Between the two LANAs, the regions of highest structural conservation contain the entire β-barrel and the helices α2 and α3.

Several amino acid residues of kLANA have previously been identified to be crucial for specific viral DNA binding [24], [45]. These are all located on the bottom part of the LANA CTDs as shown in Figures 1C and S1C (see also Figure S4C). For example, the peptide segment PYG at position 1065–1067 of kLANA forms a solvent accessible epitope at the beginning of helix α2 (Figure 1C). As shown previously, substitution of these three residues to alanines abolishes binding of LANA to the LBS sites in the KSHV TR [24], [45]. We confirmed this finding by electrophoretic mobility shift assay (EMSA) using a dsDNA oligonucleotide probe containing LBS1 and LBS2 (Figure S1F). We additionally tested the single point mutants S1086A and S1086E which remove a single OH group, or add a single negative charge in the center of the expected DNA binding site, respectively. As expected, S1086E strongly reduced specific DNA binding and therefore also impaired latent replication, whereas S1086A had no effect on either of these functions (Figure S1F,G). Thus, consistent with the previously reported EBNA-1:DNA complex structure [42], our findings demonstrate that the ‘bottom’ face of the kLANA CTD mediates the specific binding to LBS1/2 in the TR region of the KSHV genome.

### Oligomerization of Dimeric LANA C-Terminal Domains

**Figure 2:**
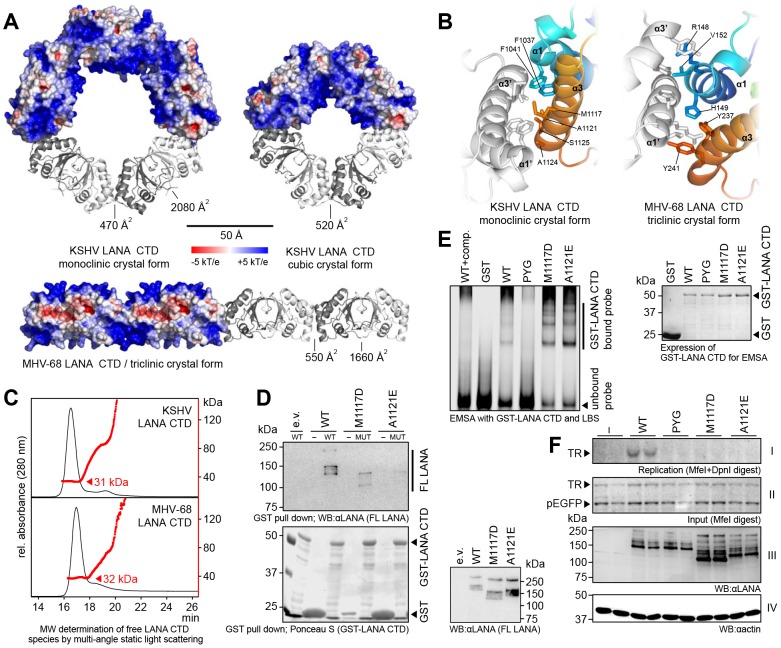
Oligomerization of the KSHV and MHV-68 LANA CTDs. **A**: Oligomeric assemblies of kLANA (top) and mLANA (below) CTD dimers as found in the respective crystals. Inter-chain contact areas within and between dimers are indicated. **B**: Details on the oligomerization sites of kLANA (left) and mLANA CTDs (right), viewed from the center of the ring (kLANA, monoclinic crystal) and the top of the linear chain (mLANA). Color scheme corresponds to Figure 1A. **C**: Flow profiles (black graph) and molecular weight (red graph) of kLANA(1013-1149) (top) and mLANA(124-260) (bottom) in asymmetric field flow fractionation. **D**: Oligomerization assay with kLANA mutants. Top left: Western blot detecting FL kLANA wt or mutants bound to GST-fused kLANA wt or mutant CTDs. Bottom left: Ponceau S –stained WB membrane showing GST-LANA(934–1162) used in this assay. (e.v.) empty vector, (-) GST only, (MUT) mutant GST-LANA CTD always corresponding to the FL LANA mutant indicated above. Right: Expression of FL LANA proteins in eukaryotic cells. The aberrant running behavior of some mutants might be due to different posttranslational modification. **E**: EMSA with LBS1+2 oligonucleotide and GST-LANA(934-1162) oligomerization deficient mutants. (wt+comp.) control with 10× excess of unlabeled probe. Right: Expression of GST-LANA CTD proteins; Coomassie stained SDS PAGE gel. **F**: Transient replication assay with oligomerization mutants and pGTR4 vector in HeLa cells. Panel I: Southern blot of replicated DNA, remaining after digest with MfeI and DpnI. Panel II: Southern blot of input DNA linearized with MfeI; pEGFP does not replicate and serves as an internal control. Assay was performed in duplicates. Panel III: LANA expression. Panel IV: Actin loading control. (-) empty vector control.

During our crystallization trials, we obtained three different crystal forms of the kLANA CTD, all of which contained higher order oligomers (Figure 2A, Table S1). The monoclinic and orthorhombic crystals of the kLANA CTD contained pentameric rings of dimers, whereas the cubic crystals contained tetrameric rings of dimers. Also mLANA CTD was observed to self-associate laterally where the dimers were arranged as linear chains. In all structures the oligomerization interfaces are formed by helices α1 and α3 (Figure 2B), and the interface comprises an area of approx. 500 Å^2^. In kLANA, the interface is mostly hydrophobic, whereas it has a more hydrophilic character in mLANA. While it was previously suggested that LANA might form native higher order oligomers [7], no equivalent intermolecular contacts were observed in any of the previously described crystal structures of EBNA-1 [42]. We thus explored if the observed LANA oligomerization interfaces are functionally important and also mediate LANA self-association *in vivo*.

We first determined the oligomerization state of recombinant kLANA(1013–1149) and mLANA(124–260) in solution. Separation of LANA CTD species by asymmetric field flow fractionation and multi-angle static light scattering showed that the purified CTDs exist mostly as dimers of 31–32 kDa in solution (Figure 2C). No monomers were observed. In order to investigate the potential existence of oligomers of LANA dimers under more physiological conditions, recombinant, immobilized GST-fused kLANA(934–1162) was incubated with extracts of eukaryotic cells containing transfected full-length (FL) kLANA(1–1162). The interaction of the two LANA proteins, indicative of self-association, could readily be detected for wt kLANA. The substitution M1117D reduced and the substitution A1121E abolished kLANA oligomerization (Figure 2D). While LBS binding was comparable to wt kLANA (Figure 2E), all lateral association mutants proved to be defective in their ability to replicate a plasmid containing four TR elements (Figure 2F). Notably, the lateral self-association of kLANA dimers appears to be independent of specific DNA binding, since kLANA CTD mutants defective in DNA binding are capable of self-association (Figure S1H). Thus, self-association of kLANA dimers *via* intermolecular side chain contacts between helices α1 and α3 is a prerequisite for the latent replication.

### Specific Interaction between LANA and ET Domains

**Figure 3:**
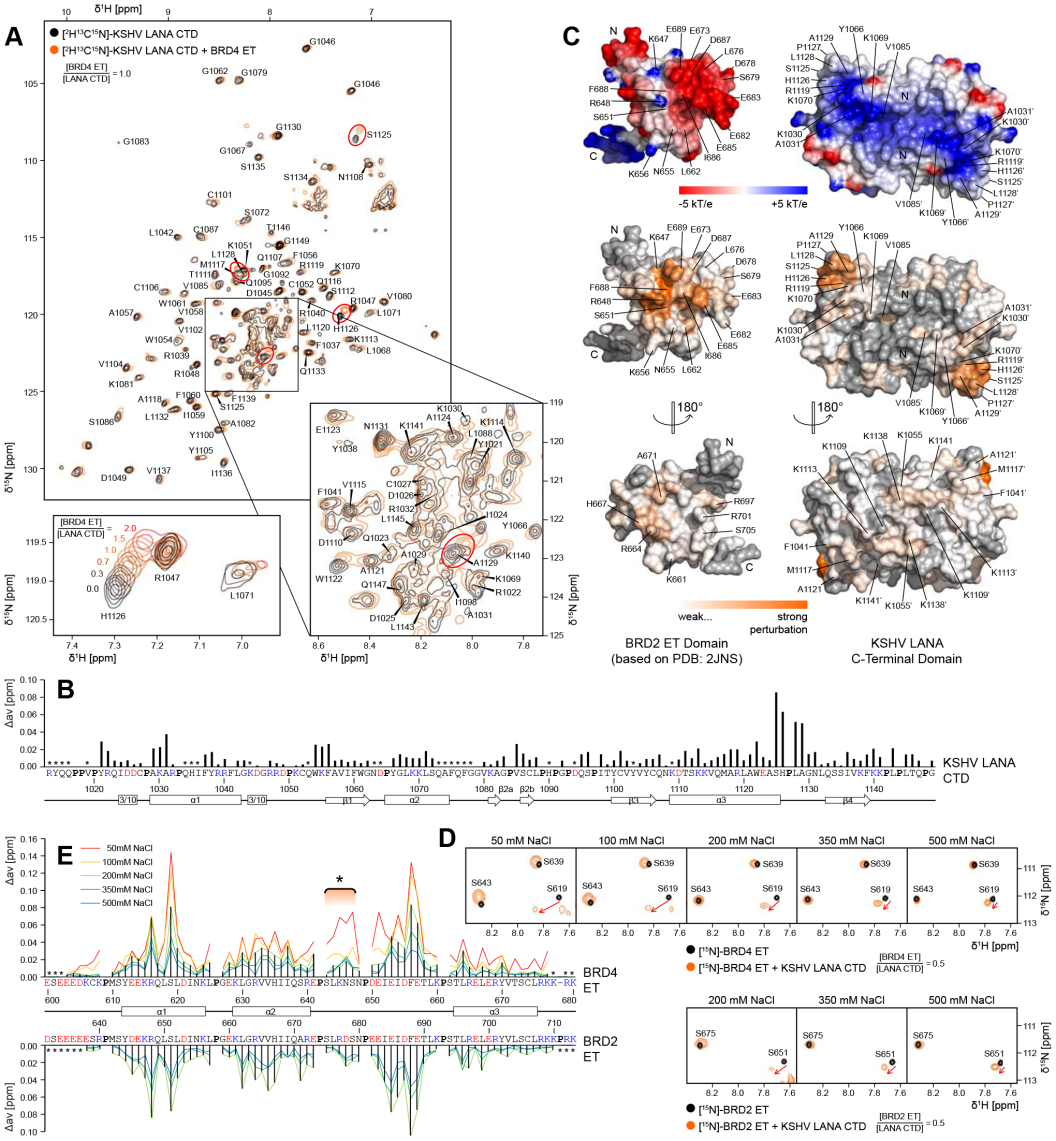
Interaction of the KSHV LANA CTD with ET Domains in Solution. **A**: [^1^H,^15^N]-TROSY spectra of 0.48 mM [^2^H,^13^C,^15^N]-kLANA(1013–1149) in 30 mM NaCl in the absence (black) and presence (orange) of 0.48 mM unlabeled BRD4(600–680). Prominent chemical shift perturbations are encircled in red. Bottom left: Chemical shift perturbation of H1126 upon titration of BRD4 ET. Bottom right: Backbone amide assignment of the central spectral region. **B**: Magnitude of chemical shift perturbations from (A) over the sequence of kLANA(1013–1149). Unassigned residues are indicated (*). Acidic residues are red and basic residues are blue, prolines are in bold face. **C**: Top: Surface electrostatic potential of BRD2 ET (left) and the kLANA CTD in bottom view (right). Below: Chemical shift perturbations mapped on the structures of BRD2 ET (200 mM NaCl) and the kLANA CTD (30 mM NaCl). Prolines and other unassigned residues are grey. **D**: Details of [^1^H,^15^N]-TROSY spectra of 0.48 mM ^15^N-BRD4(600–680) (top) and ^15^N-BRD2(632–713) (bottom) at different NaCl concentrations in the absence (black) and presence (orange) of 0.96 mM unlabeled kLANA(996–1153). Perturbation of S619 (BRD4) and S651 (BRD2) is indicated by an arrow. **E**: Magnitude of chemical shift perturbations from (D) over an alignment of BRD4 (top) and BRD2 (bottom) ET domains at different NaCl concentrations. Histogram bars are given for 200 mM NaCl. A region of strong chemical shift perturbation only at 50 mM NaCl is indicated (*); compare Figure 4D.

We and others have previously shown that the ET domains of BRD2 and of BRD4 interact with kLANA and mLANA [26], [27], [28], [40]. In order to determine the specific interaction sites in the kLANA CTD and the ET domains of BRD2 and BRD4, we performed chemical shift perturbation experiments by nuclear magnetic resonance (NMR) spectroscopy. [^2^H,^13^C,^15^N]-labeled kLANA(1013–1149) yielded a well dispersed [^1^H,^15^N]-TROSY spectrum that is characteristic of a folded globular protein (Figure 3A). When we added unlabeled BRD4 ET(600–680) to [^2^H,^13^C,^15^N]-labeled kLANA(1013–1149), only some of the kLANA resonances shifted significantly in an ET domain concentration-dependent manner (Figure 3A). The detailed quantification of the chemical shift perturbations (Figure 3B) revealed the kLANA peptide segment 1125–1129 at the C-terminal end of helix α3 to be most affected, and there were no significant alternative epitopes identified (Figure 3C, right). Thus, BRD4 ET(600–680) binds to a single site on the kLANA CTD surface.

In order to identify the binding site for the kLANA CTD on the ET domains of BRD2/4, we performed the complementary experiment. [^1^H,^15^N]-TROSY spectra of either [^1^H,^15^N]-labeled BRD2 ET(632–713) or BRD4 ET(600–680) were recorded in the absence and in the presence of unlabeled kLANA(996–1153) (Figures 3D,E). The ET:kLANA interaction strongly depends on charge, which is evident from the ionic strength dependent chemical shift perturbations (Figure 3E). The NMR line-broadening follows the same trend for all observed peaks (Figure 3D), which suggests an overall increasing particle size upon strong ET:kLANA association. The most affected backbone amide groups in the BRD4 ET domain comprise R616, S619 and E653–E657 (Figure 3E, top). The interaction with BRD2 ET(632–713) was observed to be virtually identical and to affect the corresponding amino acids R648, S651 and E685–E689 (Figure 3E, bottom). When mapped onto the surface of the globular ET domain [46], all of these affected amino acids are contiguous and form a single epitope (Figure 3C, left). Thus, the kLANA CTD binds at a single site on surfaces of BRD2/4 ET domains.

**Figure 4:**
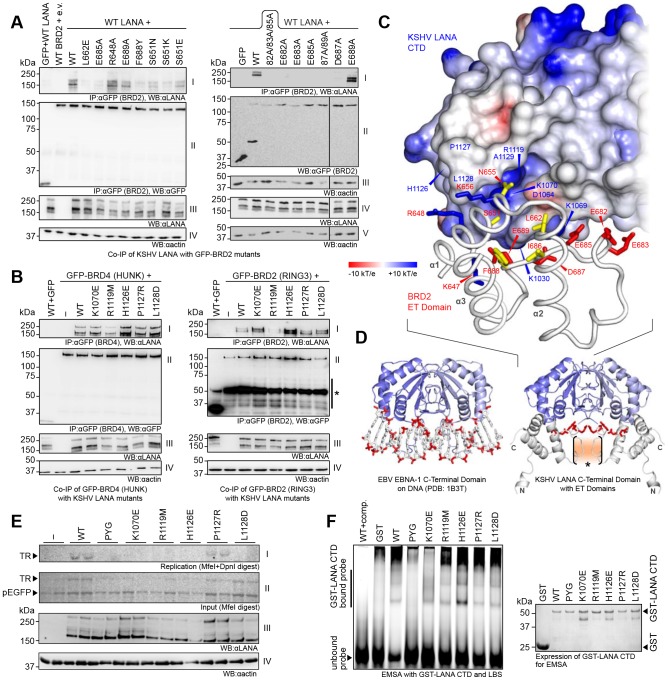
Interaction of the KSHV LANA CTD with ET Domains *in vivo*. **A**: kLANA wt was co-immunoprecipitated with GFP-tagged full-length BRD2 wt or mutants. Left: Panel I: Immunoblot of co-IP samples detecting LANA. Panel II: Blot of the same samples detecting GFP-BET proteins. Panel III: Expression of LANA in all of the samples. Panel IV: Actin loading control. Right: Immunoblot of co-IP samples with αLANA antibody (panel I), blot of the expression of the GFP-BRD2 proteins (panel II) and of LANA (panel IV) in all of the samples. Panels III and V: Actin loading control for GFP-BRD2 and LANA expression, respectively. ‘82A/83A/85A’: Triple mutant E682A/E683A/E685A, ‘87A/89A’: Double mutant D687A/E689A. **B**: kLANA ‘ET binding site’ mutants were co-immunoprecipitated with GFP-tagged BRD4 (HUNK; left) and BRD2 (right). Panels as described for (A) left side. (*) nonspecific bands appearing with some αGFP antibody lots. **C**: Detail of the complex ‘model II’ of kLANA (surface electrostatic potential representation) and BRD2 ET (cartoon representation). **D**: DNA-bound EBNA-1 CTD (left) and ‘model II’ of the kLANACTD:ET complex (right) in comparison. The acidic DNA backbone and the acidic loop region of the ET domains are shown in red. The region of strong chemical shift perturbation only at 50 mM NaCl is indicated (*). **E**: Transient replication assay with ‘ET binding site’ mutants and pGTR4 vector in HeLa cells. Panels I–IV as described in Figure 2F. **F**: EMSA with LBS1+2 oligonucleotide and GST-LANA(934–1162) wt or ‘ET binding site’ mutants. (wt+comp.) control with wt LANA and 10× excess of unlabeled probe. Right: Expression of GST-LANA CTD proteins; Coomassie stained SDS PAGE gel. See also Figure S2.

In order to confirm the binding site for kLANA on the ET domain we generated mutations in the ET domain of FL GFP-BRD2 and tested them in co-immunoprecipitation experiments with FL wt kLANA (Figure 4A). The alanine substitution of any one of the negatively charged amino acids contained in the peptide segment BRD2-ET(682–687) resulted in the complete loss of kLANA binding (Figure 4A). Consistent with our NMR data showing ionic strength-dependence of kLANA:ET binding, this result confirms the importance of electrostatic forces in this interaction. The F688Y substitution also abolished binding to kLANA. The substitution of S651 to any larger amino acid severely reduced the interaction with kLANA presumably by steric effects that appear to dominate there. Only the R648A and E689A substitutions appeared not to affect kLANA binding. Thus, most amino acid substitutions in the vicinity of the kLANA binding epitope of BRD2 ET reduced or abolished binding, confirming the interaction site as identified by NMR.

We also generated a panel of mutants of FL kLANA and tested them in a co-immunoprecipitation assay with GFP-tagged BRD2 and BRD4 (Figure 4B). The kLANA R1119M substitution impaired binding of the ET domains of both BET proteins. R1119 forms a positive charge at the bottom of a cleft, which is occupied by anions in the kLANA crystals (Figure S2B). The substitution at this position might thus abolish anion binding, rearrange the adjacent peptide segment 1125–1129 and reduce ET binding. The substitutions P1127R had no significant effect on ET binding, while the mutations H1126E and to a lesser degree L1128D, enhanced BET protein binding. The results were similar for BRD2 and BRD4 suggesting that the mode of interaction is virtually identical for both proteins. We next compared the binding of these kLANA mutants to FL BRD2, the BRD2 ET domain alone (ET: aa640-719) or the entire C-terminal domain of BRD2 (CT: aa640-801). We found that the binding of the BRD2-ET to LANA was much more susceptible to the LANA substitutions H1126E, L1128D, R1119M then binding of the FL BRD2 or the BRD2-CT (Figure S2B). This result indicates that, while the ET domain binds to the kLANA region defined by aa 1125–1129, an additional contact point may exist in the BRD2 C-terminal domain or FL BRD2 (see also below).

To further delineate regions in kLANA that contribute to its interaction with BET proteins, four double-point charge inversion substitutions were selected in a reasonable radius around the peptide segment 1125–1129 of the kLANA CTD. The mutants were tested for their ability to induce specific chemical shift perturbations in ^15^N-labeled BRD4-ET(600–680) (Figure S2C). The substitutions KK(1113–1114)EE and KK(1140–1141)EE did not disrupt binding, but the substitutions KAR(1030–1032)EAE and KK(1069–1070)EE, located at the ‘bottom’ of the LANA structure that mediates the specific binding to viral DNA (see above) led to significant impairment. Based on these data we performed *in silico* docking of the globular ET domain to kLANA using ‘Rosetta Dock’ [47]. Two global orientations of BRD2 ET(632-713) relative to kLANA(1013–1149) displayed comparably low energy scores (Figure S2D). In both models, F688 of BRD2 ET was placed near the N-terminus of helix α2 of kLANA. In model I, the acidic peptide segment of residues 682–689 in BRD2 ET is placed in the basic cleft of kLANA right underneath the peptide segment 1125–1129. In model II (Figure 4C, S2D), BRD2 ET is rotated by 180° relative to model I. Notably, in our NMR study we initially observed particularly strong chemical shift perturbations only at low salt concentrations in the segment 643–647 of BRD4 ET(600–680), (Figure 3E). This structural element of ET forms a surface exposed loop between helix α2 and the major LANA binding epitope of ET. Since LANA is a dimer, two ET domains can bind to it. However this only occurs to a significant degree at low salt concentrations, when electrostatic interaction is strong. In model II this would lead to cooperative self-interactions at the above-mentioned segment of ET (Figure 4D, S2D). Thus, only the second model is supported by the observed salt-dependent NMR chemical shift perturbations and is also in better overall agreement with the corresponding mutagenesis data.

We next investigated how the ET binding site in the kLANA CTD contributes to kLANA's functions (Figure 4E, F). All mutants in the ET binding site, except for P1127R, were incapable of replicating a plasmid carrying 4 TR elements in transiently transfected cells (Figure 4E). With the exception of K1070E all mutants were still able to bind LBS (Figure 4F). The ability of kLANA ET binding site mutants to oligomerize was comparable to wt kLANA (Figure S2E). Thus, several of the kLANA residues whose NMR resonances shift in response to ET domain binding are required for successful latent episomal replication without affecting the binding to the viral latent origin of replication or kLANA oligomerization.

### The ‘Basic Top’ in LANA Function

**Figure 5:**
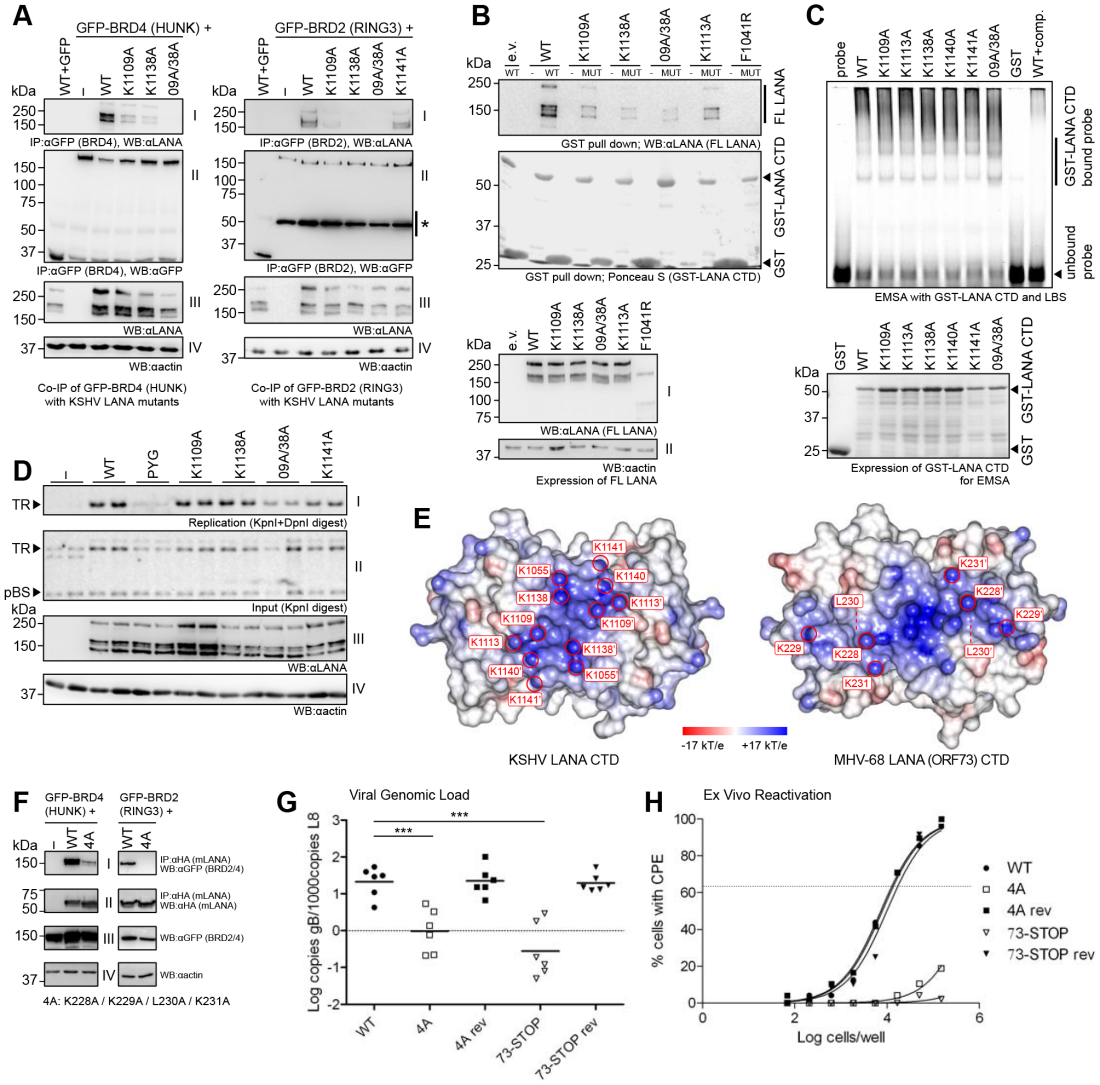
Role of the ‘Basic top’ of LANA in BET Protein Interaction and Oligomerization. **A:** Co-IP of kLANA ‘basic top’ mutants with GFP-BRD4 (left) and GFP-BRD2 (right). Panels I–IV as in Figure 4B. ‘09A/38A’: K1109A/K1138A mutant. **B:** Oligomerization assay with kLANA ‘basic top’ mutants. Upper top: WB detecting FL kLANA wt or mutants bound to GST-kLANA wt or mutant CTDs. Lower top: same WB membrane as above detecting GST-LANA CTD proteins. (e.v.) empty vector, (-) GST alone, (MUT) mutant GST-LANA CTD corresponding to the FL LANA mutant indicated above. Bottom: Expression of FL LANA proteins and the actin control. **C:** EMSA with LBS1+2 oligonucleotide and GST-LANA(934-1162) ‘basic top’ mutants. (wt+comp.) control with 10× excess of unlabeled probe. Below: Expression of the GST-LANA CTD proteins (Coomassie stain). **D:** Transient replication assay with kLANA ‘basic top’ mutants and pTR1 vector in HeLa cells (performed in duplicates). Panel I: Southern blot of replicated DNA, remaining after digest with KpnI and DpnI. Panel II: Southern blot of input DNA linearized with KpnI; pBluescript (pBS) does not replicate (internal control). (-) empty vector control. Panel III: LANA expression. Panel IV: Actin control. **E:** Top view of kLANA and mLANA CTDs. Mutated residues are labeled. **F:** Co-IP of GFP-BRD4 (left) and GFP-BRD2 (right) with HA-mLANA wt or 4A mutant. Panels I: Immunoblot of co-IP samples detecting GFP-BET proteins. Panels II: Blot of the same samples detecting HA-mLANA. Panels III: Expression of BET proteins. Panels IV: Actin loading control. **G:** C57BL/6 mice were infected i.n. with the MHV68 wt, the mLANA: 4A (‘4A’) and STOP (‘73-STOP’) mutant viruses and respective revertants (‘4A rev’), (‘73-STOP rev’). DNA isolated from splenocytes was used for qPCR analysis. Marks represent individual mice and the bars the means. Data include two independent experiments. (***) P<0.001. **H:** *Ex vivo* reactivation assay with the splenocyte samples from (G). Dashed line: the point of 63.2% reactivation (MOI = 1).

Although the fold of the two γ2-herpesviral LANA CTDs is closely similar to that of the γ1-herpesviral EBNA-1, their surface charge at the face opposing the specific DNA binding site is inverted (Figure 1B). The conservation of the basic, lysine-rich patch in the top surfaces of both kLANA and mLANA may indicate an important, conserved function (Figure S4C, S1D). Indeed, the alanine substitution of two single lysine side chains K1109 and K1138 severely reduced both BRD2 and BRD4 binding, while the double substitution K1109A/K1138A completely abolished it (Figure 5A). While the substitutions K1055A and K1113A did not significantly affect BRD4 binding, they abolished BRD2 binding by kLANA (Figure S3A). As we already mapped a specific ET domain interaction to the 1125–1129 epitope in kLANA, this result suggested that a second significant interaction site between LANA CTD and BET proteins exists.

An amino acid sequence alignment of the BRD2/4 ET domains including their C-terminally adjacent sequence elements pointed to an acidic, serine-rich stretch as a candidate for this interaction (Figure S3B). In order to test this hypothesis, a range of N- and C-terminally truncated fragments of BRD2(640–801) was generated and assayed for their capability to interact with FL kLANA (Figure S3C). Binding of BRD2(640–801) was very strong, while the fragment BRD2(720–801), which lacks the globular ET domain, did not bind kLANA. Likewise, the fragment BRD2(640–773), which only lacks the serine-rich sequence element, was severely impaired in kLANA binding. Using NMR spectroscopy, we observed that the ^15^N-labeled BRD4(600–722), which contains the ET domain and the C-terminal serine-rich peptide, behaves closely similar to ^15^N-BRD4-ET(600–680) in solution (Figure S3D). However, when kLANA(996–1153) was added, resonances broadened substantially and the spectrum of BRD4(600–722) nearly disappeared, indicating a substantially increased particle size and strong binding. This effect was considerably milder when kLANA(996–1153) was added to ^15^N-labeled ET fragments lacking the serine stretch. Thus, two independent experimental approaches show that the serine-rich acidic sequence of BRD2/4 strongly enhances kLANA interactions with BRD2/4.

In order to further understand the role of the kLANA ‘basic top’, we tested mutants of this area with respect to other LANA functions. The pull down of full-length LANA with a GST-fused LANA CTD was impaired by lysine to alanine substitutions in this region. In particular, K1109A/K1138A severely reduced kLANA oligomerization (Figure 5B). None of these lysine to alanine replacements affected binding to LBS DNA (Figure 5C). However, in the transient replication assay (Figure 5D) the double mutant (K1109A/K1138A) showed a consistently reduced ability to replicate latent viral DNA, while the corresponding single substitutions had no significant effect.

We have previously shown that residues 228–231 (KKLK), which are located on the basic surface of mLANA (Figure 5E), influence the interactions with BRD2 and BRD4, and affect mLANA-mediated transcriptional activation [40] (see also Figure 5F). In order to assess the functional role of this basic surface element *in vivo*, we inserted this mLANA mutant into a recombinant MHV-68 genome cloned into a bacterial artificial chromosome. Mutant and revertant viruses were then used to infect mice intranasally and compared to a previously described [36] mLANA deletion mutant (‘73-STOP’) and its revertant. The 228–231 4A mutant replicated normally in tissue culture (not shown). On day 6 after intranasal infection, this mutant replicated to wt levels in the lungs of infected mice (not shown). However, on day 17 after infection, when MHV-68 latency had been established, the 4A mutant showed a pronounced reduction of viral genome copy numbers in spleen cells approaching the phenotype seen with the mLANA deletion mutant (Figure 5G). In a reactivation assay with latently infected splenocytes plated on a susceptible cell line, even high numbers of splenocytes failed to yield reactivatable virus (Figure 5H). Based on these data, we conclude that the ‘basic top’, common to kLANA and mLANA proteins, plays an important role in the interaction with the BET proteins and contributes to latent replication/persistence.

### The Contribution of LANA CTD Surfaces to Nuclear ‘Speckle’ Formation

**Figure 6:**
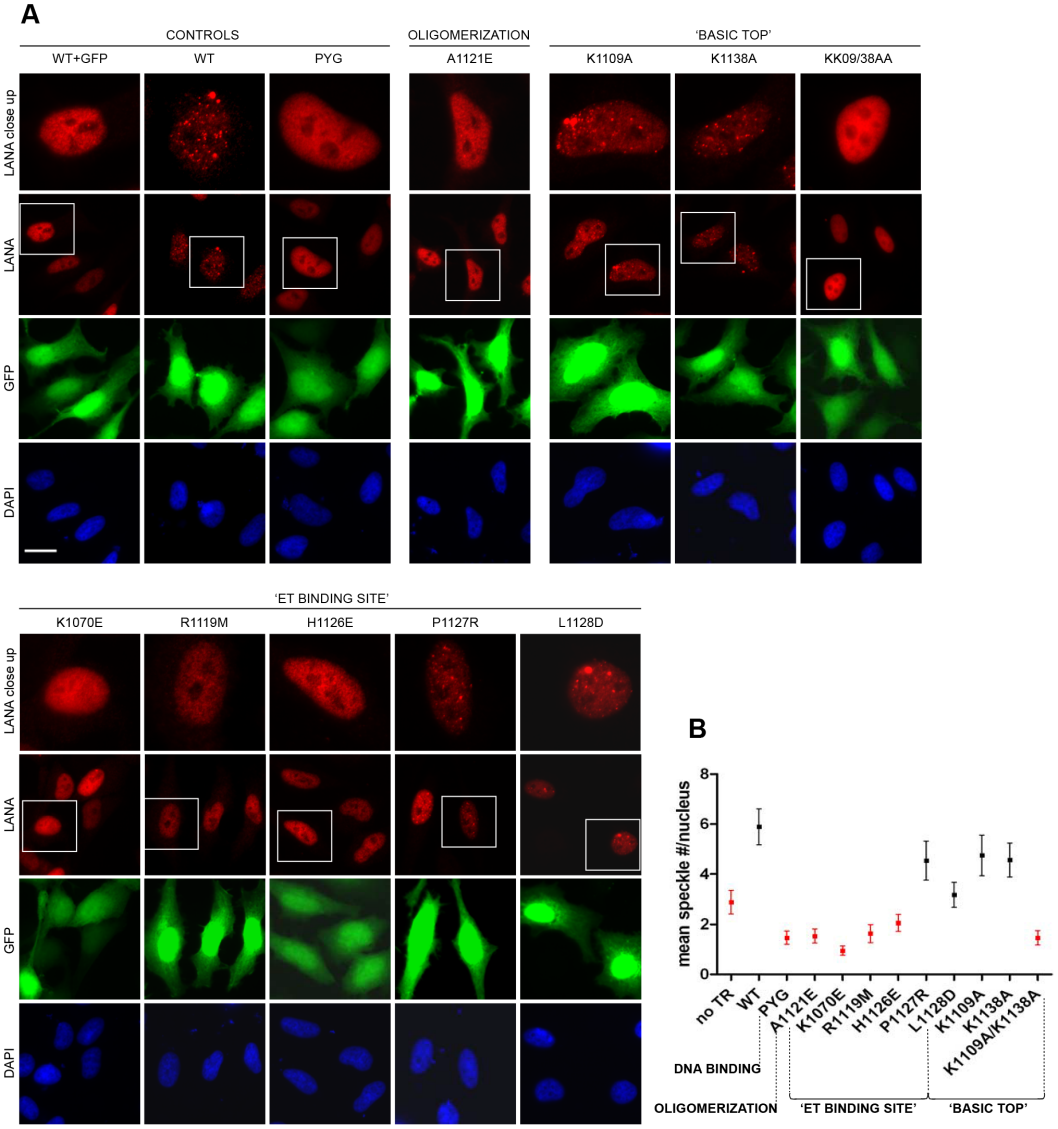
Contribution of Different Sites in KSHV LANA CTD to the Formation of LANA Nuclear ‘Speckles’. A: Speckle formation assay with kLANA mutants of different sites. HeLa cells were transfected with vector containing 4xTR and kLANA wt or mutant vectors. kLANA was stained with a mouse αLANA antibody and DNA was stained with DAPI. Expression of GFP confirms the presence of the TR containing vector in the cells. Close up of an exemplary single cell from each of the images is shown in the top row. Images taken at 63× magnification, a scale bar of 20 µm is shown in the DAPI image of the first sample (wt+GFP). **B:** Quantification of the number of LANA speckles per nucleus performed with Cell Profiler software. The mean LANA speckle number per nucleus is plotted on the graph for each of the LANA proteins. A total of 80–110 cells were analyzed per sample in two independent experiments. The error bars indicate SEM. Speckle numbers in the samples marked in red are significantly different from those obtained with wt LANA.

One of the characteristic features of kLANA is the formation of nuclear micro-domains (‘speckles’) in latently infected cells [3], [48], [49]. The viral genomes colocalize with LANA in these speckles, and the presence of the KSHV TR region, which contains the LBS sites, is required for their assembly [19]. We tested a selection of the kLANA mutants described above for their ability to form nuclear speckles in the presence of a TR containing plasmid (Figure 6A). The FL wt kLANA protein formed many distinct speckles in most of the transfected cells, while the LBS binding deficient PYG(1065–1067)AAA mutant (Figure S1F), showed a diffuse nuclear localization, indicating that specific viral DNA binding is a prerequisite for kLANA speckle assembly. Also the kLANA mutant A1121E, defective in self-association (Figure 2), did not form nuclear speckles suggesting a crucial role of inter-dimer interactions between helices α1 and α3 in this process. Individual substitutions of lysine residues on the ‘basic top’ of kLANA did not result in a loss of speckles, while the double mutant K1109A/K1138A showed an impairment of speckle formation (Figure 6A,B). This indicates that also the ‘basic top’ contributes to higher order oligomerization of LANA *in vivo*. Furthermore, mutants K1070E, R1119M and H1126E localized near the ET binding site were compromised in speckle formation, while the surface exposed residues P1127 and L1128 did not significantly affect this process (Figure 6A,B). Overall, the sites for viral DNA binding, self-association of kLANA dimers, and residues near a binding site for chromatin-associated BRD2/4 proteins are required for formation of the characteristic kLANA nuclear speckles.

## Discussion

In this study we solved the 3D structures of kLANA CTD and mLANA CTD. They share the overall fold with the origin binding proteins of Epstein-Barr virus, EBNA-1 [42] and of human papillomavirus, E2 [43]. However, their surface characteristics are substantially distinct from EBNA-1 and E2, which provided the basis for a specific functional annotation of three faces in the CTDs of kLANA and mLANA (see also Table S2).

The self-association of LANA CTDs observed in both kLANA and mLANA crystals represents a key characteristic feature of these proteins. In all crystals, the inter-dimer interactions were mediated by helices α1 and α3. The angles between two neighboring LANA dimers range from 0° for mLANA, 72° for the kLANA pentameric rings to 90° for the kLANA tetrameric ring (Figure 2A,B). Moreover, both pentameric ring structures of kLANA dimers display ring puckering, where the interface between two dimers is oriented out of the ring plane (Figure S4B). This provides direct crystallographic evidence that the interface between LANA dimers is generally compatible with a range of relative LANA dimer orientations. Due to the hinge-like character of the inter-dimer interface, *in vivo* native LANA oligomers might differ in shape from the closed ring structures or the linear chain observed in the respective crystals.

EBNA-1 dimers physically link target viral DNA sites *via* their linking domains that are distinct from the DNA binding domains [50]. The ability of EBNA-1 to self-associate through disparate domains was shown to be important for its function in viral replication and transcriptional control [51]. In contrast to EBNA-1, self-association of LANA appears to be mediated by its C-terminal DNA binding domain.

kLANA binds cooperatively to LBS1 and LBS2 within the KSHV TR [4]. DNA bending has been proposed to be a prerequisite for the initiation of DNA replication [52], [53] and a LBS1/2 containing probe was previously shown to be bent by ∼110° when both sites were occupied [54]. Two adjacent kLANA dimers in our monoclinic crystals are perfectly oriented to bind and bend LBS1/2 to this extent (Figures 2A, S4A). While our oligomerization mutants of kLANA (M1117D and A1121E) could still bind DNA (Figure 2E) they were not able to promote KSHV episome replication (Figure 2F). Consequently, kLANA inter-dimer interactions may contribute to KSHV latent replication through their impact on DNA bending.

On the other hand, self-association of kLANA dimers appears to be functionally important beyond the bending of viral DNA, because mutations that disrupt the inter-dimer interface also abolish the formation of the characteristic nuclear kLANA speckles (Figure 6). Unexpectedly, we found that also the positively charged ‘top’ of the kLANA CTD contributes to speckle formation. In particular the double mutant K1109A/K1138A, which removes four positive charges per kLANA dimer, shows reduced oligomerization (Figure 5B) and speckle formation (Figure 6). Although this mutant was not impaired in LBS binding, it showed a reduced capacity to replicate a TR containing plasmid (Figure 5C,D). Likewise, the mLANA 4A mutant was incapable of establishing latency in the spleens of infected mice (Figures 5G,H). Thus, interaction partners other than LBS1/2 appear to significantly enhance LANA speckle formation *via* the ‘basic top’, which appears to be generally required for latent replication/persistence.

We found that the binding of BET proteins to kLANA occurs *via* two distinct sequence elements of BRD2/4. In addition to the interaction of the serine-rich stretch of BET proteins with the ‘basic top’ of LANA CTD, their globular ET domain binds near residues 1125–1129 of kLANA (Figures 3, 4, S4C). Both interactions are required for strong LANA:BET binding (Figure S3C). The serine-rich tails of BET proteins might interact with neighboring LANA dimers, which would stabilize LANA oligomers. This interaction may therefore promote the oligomerization of LANA CTDs and consequently the formation of nuclear speckles.

**Figure 7:**
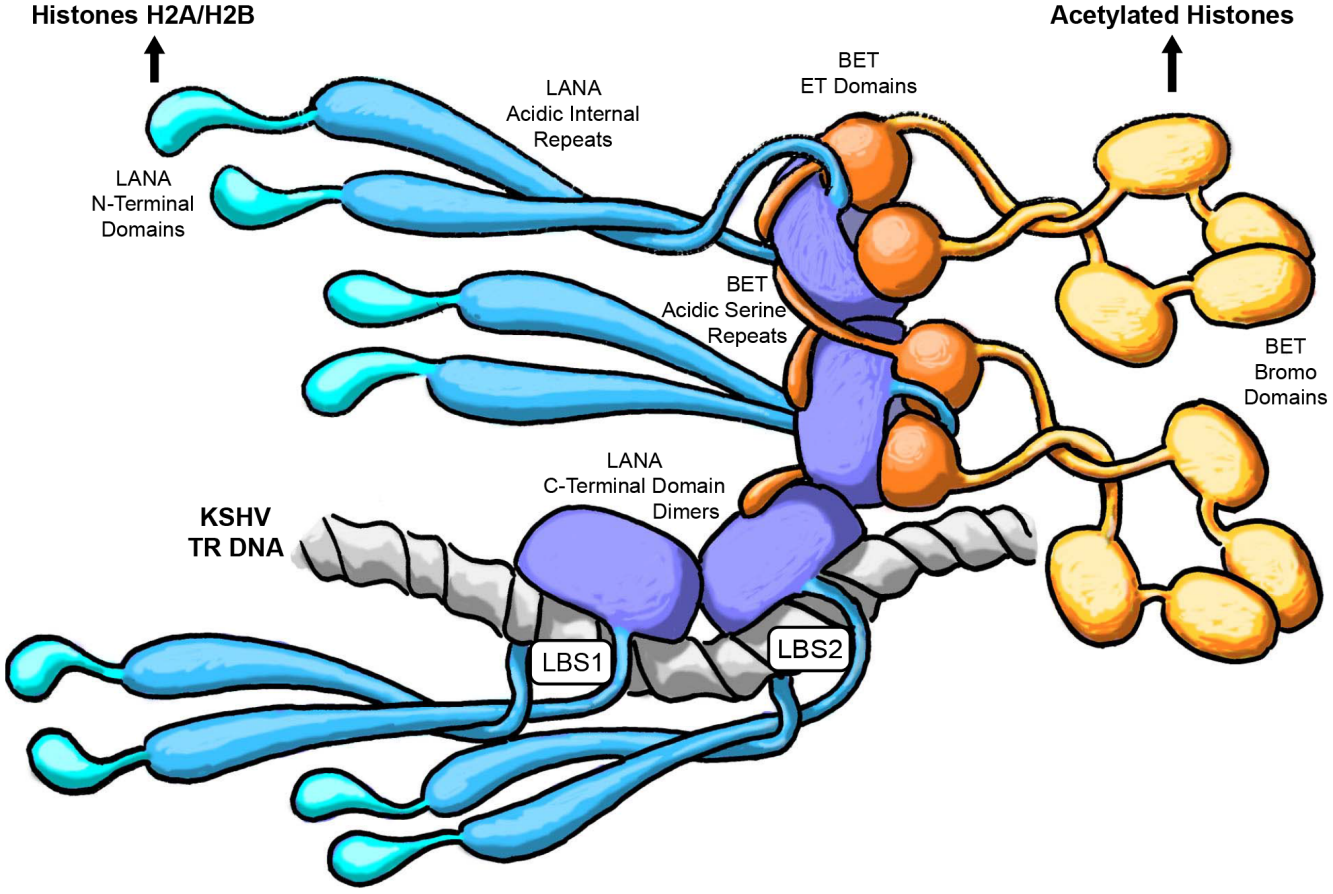
Hypothetical model of KSHV LANA oligomers. Dark blue: kLANA CTD dimers. Cyan: kLANA acidic internal repeat regions and N-terminal domains. Grey: DNA. Orange: BET proteins.

In solution, the kLANA and mLANA CTDs exist as isolated dimers (Figure 2C) suggesting that other interaction partners of LANA might be required to shift the equilibrium towards higher order LANA oligomers in order to promote speckle formation *in vivo*. However, for sterical reasons pairs of LBS1/2-bound kLANA dimers could not oligomerize directly, but would require at least one more bridging dimer between them. Interaction of such LBS-free LANA dimers with LBS-bound dimers could be enhanced by other binding partners like the BET proteins (Figure 7). While the present data do not allow final conclusions, we are tempted to speculate that LANA speckles contain LANA oligomers that are stabilized by interactions with (i) LBS DNA, (ii) BET proteins, and potentially (iii) other interaction partners of FL LANA. Such a scenario is consistent with our observations, and it would allow tethering many TR-repeats into a single nuclear speckle.

Our NMR data place the ET domain binding site and the viral episome binding site in very close proximity on the kLANA surface, which makes the simultaneous binding of LBS and ET on the same kLANA dimer unlikely due to sterical hindrance. However, it is well established that viral episomes and BET proteins colocalize with the LANA speckles [28], [55]. This can be reconciled in a model that envisages viral DNA and BET proteins interacting with distinct kLANA molecules that are present in the same oligomeric aggregate (Figure 7). It appears possible that in addition to the acidic serine-rich sequence element of the BET proteins, also other acidic factors might stabilize kLANA oligomers by interactions with its ‘basic top’. Candidates would also include kLANA's own acidic internal repeat region or the phosphate backbone of DNA molecules. kLANA has been shown to interact also with other host chromatin proteins [11] and thus it is conceivable that further contributors to kLANA oligomerization exist. Overall, kLANA CTD oligomerization could allow the alternative occupation of overlapping binding sites on different LANA molecules in the nuclear speckles.

The N-terminal residues 5–13 of kLANA are required for chromatin association [22], [25], [56] and latent replication [57]. A chromatin binding domain has also been identified in the kLANA CTD [23], [24], [58]. Previous studies found that multiple alanine substitutions near residues 1125–1129 of the kLANA CTD abolish its association with the mitotic chromosomes [23] (Figure S4C). We found that BET proteins, known to associate with mitotic chromosomes [59], [60], interact with this part of kLANA CTD (Figure 3A–C, 4B) and could therefore contribute to interactions between the kLANA CTD and host chromosomes. Most of our kLANA mutants targeting the ET domain binding site were impaired in latent replication/persistence (Figure 4E) and kLANA speckle formation (Figure 6). Thus, in addition to LBS DNA binding and kLANA oligomerization, kLANA chromatin association through BET proteins could be a third essential factor for KSHV persistence.

In conclusion, our structural and functional data revealed kLANA oligomerization *via* its CTD as an Achilles' heel of this γ2-herpesvirus. A single point mutation at the critical kLANA interface has the potential to abolish KSHV persistence and thus provides a possible antiviral target. In addition, the promotion of kLANA oligomerization by host protein contributors may provide further points of interference to compromise latent viral replication, and thereby prevent human diseases that are caused by persistent KSHV infections.

## Materials and Methods

### Ethical Statement

All animal experiments were in compliance with the German Animal Welfare Act, and the protocol was approved by the local Animal Care and Use Committee (District Government of Upper Bavaria; permit number 124/08).

### Bacterial Protein Expression for Crystallization and NMR Spectroscopy

All protein constructs were expressed from synthetic genes (Invitrogen) in pET-based vectors providing resistance to ampicillin. All of them carried an N-terminal his_6_ tag joined by a thrombin protease cleavage site. Expression was carried out in *E. coli* BL21(DE3) (Stratagene). Unlabeled proteins were produced in 1 L shaking flask cultures of ZYP-5052 auto-inducing rich medium [61], which were inoculated with a starting OD_600_ of 0.1 and were incubated over night at 37°C.

For expression of seleno-methionine (SeMet) labeled KSHV LANA(1013-1149), a preculture of 2×1 L minimal medium was first grown from an OD_600_ of 0.1 to 1.0 at 37°C (80 mM K/Na-phosphate pH 7.0, 40 mM NH_4_Cl, 4 mM Na_2_SO_4_, 2 mM MgSO_4_, 0.5% (w/v) glucose, 33 µM thiamine chloride, 0.2× trace metals [61].The complete cell mass was then transferred to 500 mL expression culture (same as above without trace metal mixture but containing 100 mg/L lysine, phenylalanine, and threonine, 50 mg/L isoleucine, leucine, and valine, and 60 mg/L seleno-methionine). Subsequently, expression was induced with 1 mM IPTG and the culture was incubated for 9 h at 37°C.

For expression of isotope labeled proteins for NMR analysis, precultures of 2×1 L CN-040 minimal medium were first grown from an OD_600_ of 0.1 to 1.0 at 37°C (100 mM K/Na-phosphate pH 7.0, 1 g/L NH_4_Cl, 5 mM Na_2_SO_4_, 2 mM MgSO_4_, 4 g/L glucose, 1× MEM vitamin solution (Sigma-Aldrich), 0.2× trace metals [61]. At this stage, no labeling substrates were included. The complete cell mass was then transferred to 500 mL expression cultures in CN-040, which depending on the requirements contained ^15^N-NH_4_Cl, ^13^C-glucose, and was based on deuterium oxide (all by Cambridge Isoptope Laboratories) for KSHV LANA(1013-1149). Expression was induced with 1 mM IPTG and the cultures were incubated for 9 h at 37°C.

### Bacterial Protein Expression and Purification for Crystallization and NMR Spectroscopy

His_6_-tagged kLANA and mLANA CTD fragments were expressed in *E. coli*. Bacterial cells were suspended in guanidine buffer (100 mM sodium phosphate, 10 mM tris-Cl, 6 M guanidine-Cl, 2 mM DTT, pH 8.0) and disrupted on an ultrasonic homogenizer until a clear solution was obtained. The homogenate was cleared from insoluble constituents by centrifugation at 37,000× g for 30 min. Protein originating from 10 g of wet cell mass was coupled to 5 mL of Ni-NTA Superflow beads (Qiagen).

For KSHV LANA and MHV-68 LANA constructs, an interrupted linear guanidine gradient was applied to the beads (6.0 M–1.8 M guanidine in 42 min at 1 mL/min, 1.8 M for 20 min at 1 mL/min, 1.8 M for 40 min at 0.1 mL/min, 1.8 M–0 M in 18 min at 1 mL/min). Proteins were eluted in imidazole buffer (100 mM sodium phosphate, 10 mM tris-Cl, 500 mM imidazole, 2 mM DTT, pH 5.8). For KSHV LANA and MHV-68 LANA constructs, the procedure of running the gradient and subsequent elution was repeated four times on the same beads and elution fractions were pooled and concentrated using 10 kD MWCO Vivaspin 20 spin filters (Sartorius). For BRD2 and BRD4 ET domain constructs a smooth linear guanidine gradient was applied (6.0 M–0 M in 60 min at 1 mL/min). Here, a single elution step was considered sufficient.

For all constructs, the buffer was exchanged to thrombin cleavage buffer (20 mM tris-Cl, 100 mM NaCl, 2 mM DTT, pH 8.0) by dialysis and the his_6_ tag was cleaved with thrombin (Sigma-Aldrich) at a concentration of 2 u/µmol at 22°C. After complete digest, the protease was inactivated with Complete EDTA-free Protease Inhibitor Cocktail (Roche) and removed on Benzamidine Sepharose 4 Fast Flow (GE Healthcare). For BRD2 and BRD4 ET domain constructs, the cleaved his_6_ tag was removed on nickel beads and the proteins were concentrated and transferred to their final buffers in 5 kD MWCO Vivaspin 20 spin filters (Sartorius). For KSHV LANA and MHV-68 LANA constructs the cleaved his_6_ tag was separated using 10 kD MWCO Vivaspin 20 spin filters (Sartorius) in the presence of 100 mM imidazole, pH 6.5. The proteins were subsequently concentrated and transferred to their final buffers on the same spin filters. All protein constructs harbored the non-native amino acid sequence glycine-serine at their N-terminus, which was left from the thrombin cleavage site. Identity, integrity, and purity of the proteins were confirmed by SDS-PAGE and mass spectrometry.

### Asymmetric Field Flow Fractionation and Multi-Angle Static Light Scattering

Flow fractionation experiments were carried out on a Wyatt Eclipse 3 separation system with a Wyatt Dawn Heleos-II static light scattering detector and a Wyatt Optilab rEX refractometer in conjunction with components of a Shimadzu HPLC system. A short flow channel (145 mm length, Wyatt) equipped with a 490 µm spacer on a 10 kD PLGC regenerated cellulose membrane (Millipore) was connected to the system. The detector flow was 1.00 mL/min and the cross flow was 2.00 mL/min. Each run, 40 µg of protein were loaded. Running buffer was 10 mM bis-tris-Cl, pH 6.5, 50 mM NaCl, 0.01% NaN_3_. For molecular weight determination, the refractive index was used as a measure of protein concentration.

### Crystallization

KSHV LANA(1013-1149), monoclinic crystal form: 1.5 µL of 2.0 mM protein (1.0 mM for the SeMet derivative) in 5 mM bis-tris-Cl, pH 6.5, 10 mM DTT were added to 1.5 µL of 0.1 M sodium bicine, pH 9.0, 1.0 M lithium chloride, 7% (w/v) PEG 6000. The mixture was incubated at 12°C in a hanging drop setup. Crystals grew in a few days and were cryo-protected by short soaking in 0.1 M sodium bicine, pH 9.0, 1.0 M lithium chloride, 7% (w/v) PEG 6000, 25% (v/v) glycerol.

KSHV LANA(1013-1149), orthorhombic crystal form: 1.5 µL of 0.8 mM protein in 5 mM bis-tris-Cl, pH 6.5, 200 mM LiCl, 4 mM DTT were added to 1.5 µL of 0.2 M lithium citrate, pH 7.6, 20% (w/v) PEG 3350. The mixture was incubated at 20°C in a hanging drop setup. Crystals grew in a few days and were cryo-protected by short soaking in 0.2 M lithium citrate, pH 7.6, 20% (w/v) PEG 3350, 30% (v/v) glycerol.

KSHV LANA(996-1153), cubic crystal form: 1 µL of 1 mM protein in 5 mM bis-tris-Cl, pH 6.5, 2 mM DTT was added to 1 µL of 0.1 M sodium citrate, pH 2.5, 1.5 M ammonium sulfate. The mixture was incubated at 4°C in a hanging drop setup. Crystals grew in a few days and were cryo-protected by short soaking in 0.1 M sodium citrate, pH 2.5, 1.5 M ammonium sulfate, 25% (v/v) glycerol.

MHV-68 LANA(124-260), triclinic crystal form: 1.5 µL of 2.0 mM protein in 5 mM bis-tris-Cl, pH 6.5, 10 mM DTT were added to 1.5 µL of 0.1 M sodium bicine, pH 9.0, 1.0 M lithium chloride, 7% (w/v) PEG 6000. The mixture was incubated at 12°C in a hanging drop setup. Crystals grew in a few days and were cryo-protected by short soaking in 0.1 M sodium bicine, pH 9.0, 1.0 M lithium chloride, 7% (w/v) PEG 6000, 25% (v/v) glycerol.

### Structure Determination

All datasets were collected under cryogenic temperatures either on beamline 14.2 of BESSY II of the Helmholtz–Zentrum Berlin (HZB), Germany or on our Rigaku MicroMax-007 HF rotating anode home source (Table S1). The crystallographic phase problem was solved through a single-wavelength anomalous diffraction experiment carried out with a seleno-methionine derivative of monoclinic KSHV LANA(1013-1149) at the absorption peak wavelength of selenium. Phase information was derived from the anomalous signal of the peak dataset using Phenix.autosol and an initial model was built using Phenix.autobuild, both of the Phenix software suite [62]. The initial model was then completed through iterative steps of manual building in Coot [63] and refinement against a high-resolution native dataset of monoclinic KSHV LANA(1013-1149) that had been corrected for anisotropic diffraction using the anisotropy correction server [64]. The datasets of orthorhombic and cubic KSHV LANA fragments as well as MHV-68 LANA(124-260) were phased by molecular replacement (MR) with Phenix.auto_mr [62] using a monomer of monoclinic KSHV LANA(1013-1149) as search model. The search model for MHV-68 LANA(124-260) was modified prior to MR by pruning non-conserved residues to account for structural differences arising from its low sequence identity with KSHV LANA. For orthorhombic and cubic KSHV LANA fragments, placed models instantly resulted good electron density maps and could be pursued with refinement. For MHV-68 LANA(124-260), however, the placed model had first to be improved by alternate rebuilding and relaxation with phenix.mr_rosetta [65]. Refinement was carried out for all structures in Phenix.refine [62] with restraints on bond lengths, bond angles, planarities, and chirality. Atomic B-factors were treated as being isotropic while the presence of anisotropic domain motion was acknowledged by performing TLS refinement. Refinement was stopped when R_work_ and R_free_ converged.

### NMR Spectroscopy

For mapping of the LANA-binding epitope on the ET domains, [^1^H,^15^N]-TROSY-type HSQC spectra [66] of 0.48 mM ^15^N-labeled BRD2(632-713) or ^15^N-BRD4(600-680) in 20 mM bis-tris-Cl, pH 6.5, 2 mM DTT, 5% (v/v) D_2_O, and NaCl in rising concentrations of 50, 100, 200, 350, and 500 mM were recorded at 20°C in the absence and presence of 0.96 mM unlabeled KSHV LANA(996-1153). While the backbone amide assignment for BRD4(600-680) was adapted from published data [46], the backbone assignment for BRD2(632-713) was obtained from a set of HNCO, HN(CA)CO, HNCACB, and CBCA(CO)NH experiments on a sample of 0.2 mM [^13^C,^15^N]-BRD2(632-713) in 20 mM bis-tris-Cl, pH 6.5, 5% (v/v) D_2_O, 200 mM NaCl, and 2 mM DTT at 20°C. Experiments were carried out on a Bruker Avance III 600 NMR spectrometer equipped with a cryogenic probehead. The ET domain constructs used in the determination of the LANA-binding epitope carried the four non-native amino acids glycine-serine-glycine-serine at their N-terminus, where the first two originated from the thrombin cleavage site and the further two were introduced as a spacer to the highly acidic N-terminus of the ET domains, which appears to inhibit thrombin digest *in cis*.

For mapping of the ET-binding epitope on the KSHV LANA C-terminal domain, [^1^H,^15^N]-TROSY-type HSQC spectra of 0.48 mM [^2^H,^13^C,^15^N]-KSHV LANA(1013-1149) in 10 mM bis-tris-Cl, pH 6.5, 5% (v/v) D_2_O, 30 mM NaCl, and 2 mM DTT were recorded at 37°C in the absence and presence of unlabeled BRD4(600-680) in concentrations of 0.14, 0.34, 0.48, 0.72, and 0.96 mM. A sequence specific backbone assignment for essentially all observable resonances of the KSHV LANA(1013-1149) was obtained from a set of TROSY-type 3D experiments (tr-HNCO, tr-HN(CA)CO, tr-HNCA, and tr-HNCACB) [67], [68] on a sample of 0.5 mM [^2^H,^13^C,^15^N]-KSHV LANA(1013-1149) in 10 mM bis-tris-Cl, pH 6.5, 5% (v/v) D_2_O, and 2 mM DTT at 37°C. These experiments were carried out on a Bruker AV 900 NMR spectrometer equipped with a cryogenic probehead. The BRD4 ET domain construct used here carried the non-native amino acid sequence GSGGGPGS at its N-terminus, where the first two residues originated from the thrombin cleavage site and the others were introduced as a spacer to the highly acidic N-terminus of the ET domain. For analysis of the chemical shift perturbations of ^1^H and ^15^N backbone resonances, a weighted average chemical shift change, Δav, was calculated according to Dehner et al. [69].

### Macromolecular Modeling

Docking simulations of KSHV LANA with the ET domain were done with the RosettaDock web server (http://rosettadock.graylab.jhu.edu/, access in January 2013 [47]. The server performs a local rigid body docking search and adjusts side chain conformations at the surface. Input PDB files contained both protein fragments at a distance of ∼10 Å to each other (Figure S2C).

The model of the BRD2 ET domain was generated based on the NMR structure of the BRD4 ET domain [46]. Both domains share a high sequence identity of 85%, where the point mutations affect only solvent-exposed residues and are mostly conservative in nature. Therefore, the 12 respective side chains of the BRD4 ET domain were altered by replacement for likely conformers of their equivalents of BRD2 using Coot [63]. Coordinates of the protein backbone and all other side chains were kept unchanged.

Bent DNA fragments for modeling of KSHV LANA DBD:LBS complexes (Figure S4) were generated with the 3D DART web server (http://haddock.chem.uu.nl/dna/dna.php, access in November 2012 [70].

### Cell Lines and Constructs for Biochemical Assays and *In Vivo* Studies

The HEK 293, HEK293T and HeLa cells were cultured in Dulbecco's modified Eagle's medium (Gibco) supplemented with 10% heat-inactivated fetal calf serum, 50 IU/ml penicillin, 50 µg/ml streptomycin, and 200 µg/ml L-glutamine at 37°C with 5% CO_2_.

Full length (FL) wt KSHV LANA in pcDNA3 (pcDNA3-LANA), GFP-BRD2, GFP-BRD4 (HUNK), pGTR4, and wt MHV-68 LANA (pVR1255 orf73) vectors were described previously [26], [71], [40], [58]. The GST-LANA CTD (aa934-1162) was cloned by PCR of the respective LANA fragment, using pcDNA3 LANA as a template, and inserted into the BamHI and EcoRI sites of the pGEX-6P1 vector, creating a construct with LANA CTD fused to the C-terminus of the GST protein. All of the point mutants of FL wt KSHV LANA, GST-LANA CTD (aa934-1162), GFP-BRD2, and FL wt mLANA were created by either single or multiple round site directed mutagenesis. All constructs were sequence verified. The TR1 vector was cloned by inserting a 590 bp fragment containing KSHV genomic sequence derived by partial digest of cosmid 83 [72] into the pBluescript vector (Stratagene). An 801 bp Not I digested TR fragment was introduced in a second step and checked for correct orientation by sequencing.

The MHV-68 genome cloned into a Bacterial Artificial Chromosome (BAC) [73] was used to produce recombinant virus. BAC mutagenesis was performed as described in [74]. The Orf73 sequence of approximately 1 kb and 1 kb of flanking sequence on each side were cloned into the shuttle plasmid pST-SNR (Kan resistance) using restriction enzymes SacI and XmaI. Mutations were introduced by two PCR reactions each using one of the flanking primers and a mutagenesis primer spanning the position to be mutated. Subsequently both PCR products were annealed and the resulting fragment amplified to obtain the entire 3 kb fragment for cloning into pST-SNR. Revertants for both mutant viruses were made by using the wt encoding shuttle plasmid. The BAC cassette was excised from viral genomes in REF-Cre cells and MHV-68 was produced in BHK21 cells as described in [73].

#### Flanking primers used

- XmaI-Orf73hom rev: CTT CCC GGG GGG CAT GCA TGA TAT GC
- SacI-Orf73hom for: AAA GAG CTC TCC TAG CTC CAT AGC ACA TAT AAA C

#### Primers used for mutagenesis

- Orf73 4A for: CAC AGT AGG CCA AGA CAA CCC TTG CTG CTGCTG CGG CCT GTT TCT TGT CTT CAA C
- Orf73 4A rev: GTT GAA GAC AAG AAA CAG GCC GCA GCAGCAGCA AGG GTT GTC TTG GCC TAC TGT G
- ORF73 K169A for: CAC CCC CCA ACA CAT TTT GCG TCA GCT GTT ATG TTT AGT AGC
- ORF73 K169A rev: GCT ACT AAA CAT AAC AGC TGA CGC AAA ATG TGT TGG GGG GTG
- ORF73 K224, 228A for: CTT TCA TTT GTT GAA GAC GCG AAA CAG GCC GCA AAA CTA AAA AGG
- ORF73 K224, 228A rev: CCT TTT TAG TTT TGC GGC CTG TTT CGC GTC TTC AAC AAA TGA AAG

### Co-Immunoprecipitation and Western Blotting

Extracts for co-immunoprecipitation assays were prepared from ∼1×10^6^ 293/293T cells transfected using Fugene6 (Promega) and harvested 2 days after transfection. Cells were suspended in 300 µl of lysis buffer (for KSHV LANA co-IPs: 50 mM Tris (pH 7.6), 60 mM NaCl, 0.5 mM EDTA, 1% glycerol, 0.2% NP-40; for MHV-68 LANA co-IPs: 20 mM Tris (pH 7.6), 150 mM NaCl, 1 mM EDTA, 1% TritonX100) with protease inhibitors: 1.5 µM aprotinin (Applichem), 10 µM leupeptin (Applichem), 100 µM phenylmethylsulfonyl fluoride (PMSF, Applichem), 1 µM benzamidine (Applichem), 1.46 µM pepstatin A (Applichem). The extracts were sonicated 3×10 s, cell debris was pelleted and the lysates were pre-cleared with Protein A/G beads (Amersham Biosciences) for 30 min at 4°C on a rolling platform. Protein A (for anti-GFP IP) or Protein G (for anti-HA IP) beads were incubated with rabbit anti-GFP antibody (Clontech) or rat anti-HA antibody (Roche) respectively, the incubation was performed overnight at 4°C on a rolling platform. Subsequently, the antibody conjugated beads were washed 3 times with 500 µl of respective lysis buffer. 20 µg (for anti-GFP) and 200 µg (for anti-HA) antibody was bound to 80 µl of Protein A beads (anti-GFP) or 100 µl of Protein G beads (anti-HA). Antibody conjugated beads were aliquoted, 15 µl per IP and 250 µl of cell extract was added per sample and incubated overnight at 4°C on a rolling platform. The beads were then pelleted and washed 6 times with respective lysis buffer. Samples were suspended in 5 µl of loading buffer (5 mM Tris pH 6.8), 45% glycerol, 5% SDS, 0.1% Pyronin Y, 3.5% β-mercaptoethanol), boiled and the proteins separated on an SDS-polyacrylamide gel (8%) and transferred to a nitrocellulose membrane. Proteins were detected using following antibodies: rat anti-LANA (ABI; 1∶500), mouse anti-GFP (Clontech; 1∶1000), mouse anti-HA (Roche; 1∶1000), and mouse anti-actin (SIGMA; 1∶1000) and visualized with the ECL reagent (Amersham Biosciences). The LAS3000 imager (FujiFilm) was used to capture the images of the signal.

### Oligomerization Assay (GST Pull Down)

Cell lysates for GST pull downs were prepared as for co-immunoprecipitation, but without pre-clearing step and the lysis buffer contained 150 mM NaCl. 250 µl of cell lysate were added per pull down sample. GST-LANA CTD (wt and mutant) beads were prepared ahead of time. 2 ml overnight bacterial culture were diluted 1∶10 and grown until the OD600 = 1.5 was reached, at which point GST-LANA CTD fusion protein expression was induced by adding IPTG (1 mM final concentration) and incubating the cultures for 4 h at 30°C. Subsequently, the cultures were spun down, resuspended in PBS with 0.5% NP40 and protease inhibitors and sonicated 3×30 sec on ice. The spin was repeated and 100 µl of 50% glutathione sepharose bead slurry, previously washed three times with wash buffer (PBS, 0.5% NP40, protease inhibitors and 5% glycerol), were added per sample and incubated overnight, while rolling at 4°C. Next, the GST fusion protein coupled beads were washed three times with wash buffer. The amount of each protein bound to 10 µl beads was estimated based on a Coomassie stain of an SDS PAGE gel and was then adjusted accordingly. After cell lysates were added to GST fusion protein coupled beads the binding reactions were incubated 3 h at 4°C on a rolling platform and subsequently washed six times with wash buffer. After adding 5 µl of loading buffer, beads were boiled and the supernatants were loaded on to a SDS-polyacrylamide gel (8%) separated and transferred to a nitrocellulose membrane. GST-LANA CTD proteins were detected with Ponceau S, directly after transfer and the full length LANA proteins with rat anti-LANA antibody, as described above for the co-immunoprecipitation.

### Electrophoresis Mobility Shift Assay (EMSA)

5′ Dy682 labeled oligonucleotides (IBA) containing both LANA binding sites

EMSA-LANA BS-top:

GAG GCG GCG CGC GGC CCC ATG CCC GGG CGG GAG GCG CCG CAG GCC CCG GCG GCG TCC CCG GC and

EMSA-LANA BS-bottom:

GCC GGG GAC GCC GCC GGG GCC TGC GGC GCC TCC CGC CCG GGC ATG GGG CCG CGC GCC GCC TC

were annealed in annealing buffer (20 mM Tris-HCl, pH 7.6, 50 mM NaCl, 10 mM MgCl_2_). GST-LANA CTD proteins (wt and mutants) were prepared as for the oligomerization assay, but following the binding to the beads and washing, they were eluted in 50 µl of elution buffer (PBS, 0.5% NP40, 1% glycerol, 60 mM glutathione, protease inhibitors, pH adjusted to 7.3) for 3 h at 4°C. The level of expression of each protein was estimated based on a Coomassie stain of an SDS polyacrylamide gel and was then adjusted accordingly. LANA proteins were incubated 30 min at room temperature in the dark, with 1 µl of 5 µM probe in a final volume of 15 µl in a buffer containing 30 mM TrisHCl, pH 7.5, 50 mM KCl, 10 mM MgCl_2_, 1 mM EDTA, 10% glycerol, 0.25% Tween 20, 1 mM dithiothreitol, 0.5 µg/µl BSA, and 0.75 µg poly(dI-dC). The samples were separated by electrophoresis on a native 4% polyacrylamide gel in 1xTris-borate-EDTA for 2 h and imaged with Odyssey Imager (LI-COR).

### Short-Term Replication Assay

This assay was performed as previously described [4]. Briefly, 1×10^5^ HeLa cells were plated per well of a 6-well plate. On the next day cells were transfected with pGTR4 plasmid [71], containing four KSHV terminal repeats (TR), and a GFP coding sequence and the pEGFP-C1 (Clontech) plasmid, used as an internal non-replicating control. Alternatively, pTR1 and pBluescript were transfected as the replicon and the non-replicating control respectively. 72 h later cells were harvested in lysis buffer (10 mM Tris-HCl pH 8.0, 10 mM EDTA, 0.6% SDS). The chromosomal DNA was precipitated overnight with 0.85M NaCl and pelleted. The episomal DNA was purified using phenol-chlorophorm extraction and the Gel lock columns (5PRIME), precipitated with ethanol and the pellet was dissolved in 20 µl of water. 90% of DNA was digested for 72 h with 60 U of MfeI HF (or KpnI) (NEB) and 60 U DpnI (NEB) and the remaining 10% with 40 U MfeI HF (or KpnI) only. The MfeI (KpnI) enzyme linearizes the pGTR4 (pTR1) as well as pEGFP-C1 (pBluescript) vectors. The MfeI (KpnI)/DpnI digestion reveals the efficiency of replication, while the single MfeI (KpnI) digestion is used to estimate the amount of input DNA. Digested DNA was loaded and separated on a 0.8% agarose gel, and transferred to a nitrocellulose membrane by Southern blotting. A sequence coding for GFP (for the pGTR4 and pEGFP) or fragment of pBluescript vector sequence (for the TR1 and pBluescript), labeled with alkaline phosphatase was used as a probe to detect the bands of interest. The AlkPhos Direct Labeling Reagents (GE Healthcare/Amersham Biosciences) were used to label the probe and the CDP-Star was used as a chemiluminescent substrate for alkaline phosphatase.

### Speckle Counting Assay

1×10^5^ HeLa cells were plated per well of a 6 well plate on coverslips and subsequently transfected using Fugene6 transfection reagent (Promega). 48 hours post transfection cells were washed with PBS and fixed with 4% paraformaldehyde (PFA) in phosphate buffered saline (PBS) (pH 7.4) for 20 min at room temperature. PFA was then quenched with 125 mM glycine and the coverslips were washed 3×5 min with PBS. Subsequently, cells were permeabilized with 0.2% Triton X-100 in PBS for 10 min at room temperature and blocked with 10% FCS in PBS for 1 h at room temperature. Next, coverslips were incubated with primary antibody (anti-LANA mouse monoclonal; 1∶100, Novocastra) in 10% FCS in PBS for 1 h at 37°C and subsequently washed 3 times with 10% FCS in PBS. The incubation with secondary antibody (donkey anti-mouse CY3 conjugated IgG, Jackson Laboratories; 1∶400) and DAPI (4′, 6-diamino-2phenylindol; 1∶100) followed for 1 h at 37°C, again in 10% FCS in PBS. Cells were again washed 3 times for 5 min in PBS at room temperature and mounted with Moviol containing DABCO.

To quantify the number of speckles per nucleus (Figure 6B) we used the CellProfiler software [75]. We analyzed minimum 80 cells per sample. All pixel intensities were rescaled to 0 to 1. Using the Otsu Global thresholding method [76] in the DAPI channel, the nuclear area was defined. Clumped nuclei were distinguished based on the shape. Next the GFP positive cells (transfected with pGTR4, which expresses GFP) were identified using the Otsu Global thresholding method distinguishing clumped cells based on intensity. Nuclei were masked with the identified GFP positive cells. Subsequently, LANA signal was masked with the nuclei of the GFP positive cells, allowing further analysis only on LANA signal from the GFP positive (and therefore also TR positive) nuclei. LANA speckles were then identified using Robust Background per object thresholding method. Clumped objects were distinguished based on intensity. Using the “Relate Objects” function we established a parent-child relationship between the LANA speckles (“children”) and the nuclei (“parents”) in order to determine the speckle number per nucleus. Total 80–110 cells from two independent experiments were analyzed per sample. Standard errors of the means and statistically significant differences were determined using Kruskal-Wallis test and Dunn's post test (GraphPad Prism, version 5.02; GraphPad Software, Inc.)

### *In Vivo* Experiments

C57BL/6 mice were purchased from Charles River Laboratories (Sulzfeld, Germany). To characterize the recombinant MHV-68 in vivo, mice were infected intranasally (i.n.) with the 5×10^4^ PFU of virus. For determination of frequency of virus reactivation and genomic load, spleens were harvested at day 17 after infection.

### Limiting Dilution Reactivation Assay

To determine the frequency of cells carrying virus reactivating from latency, serial threefold dilutions of splenocytes (starting with 1.5×10^5^ cells/well) were plated onto NIH 3T3 cells (10^4^ cells/well), as described previously [77]. The presence of preformed infectious virus was determined by plating parallel samples of mechanically disrupted cells (latent virus cannot reactivate from killed cells). A non-linear regression plot was used to infer frequencies of reactivating cells, based on the Poisson distribution, by determining the cell number at which 63.2% of the wells scored positive for a CPE (MOI = 1) – dashed line in Figure 5H.

### Measurement of Latent Viral Load by Q-PCR

Viral load in the spleens of infected mice was determined, as described previously [78] by quantitative real-time PCR using the ABI 7300 real-time PCR system (Applied Biosystems, Foster City, CA) and TaqMan universal PCR master mix (Life Technologies). Using primers and probes as described previously [79], a 70 bp region of the MHV-68 glycoprotein B (gB) gene was amplified from spleen cells DNA and viral DNA copy numbers were quantified. The murine ribosomal protein L8 (rpl8) was amplified in parallel and used to normalize between the samples. The data are presented as viral genome copy numbers relative to the copy number of L8. The quantification limit was set at 50 copies per sample, according to published recommendations [80].

The statistical analysis was performed with Graph Pad Prism 5.02 (GraphPad Software, Inc.) The P values were calculated with One Way Anova analysis with Tukey's multiple comparison test. (***) P<0.001.

### Accession Numbers

The atomic coordinates and structure factors have been deposited in the RCSB Protein Data Bank with the following accession numbers: **2YPY**: kLANA(1013–1149), decameric ring, monoclinic crystal form; **2YPZ**: kLANA(1013–1149), decameric ring, orthorhombic crystal form; **2YQ0**: kLANA(996–1153), octameric ring, cubic crystal form; **2YQ1**: mLANA(124–260), triclinic crystal form.

#### Other previously published proteins mentioned in this work

UniProt Identifiers: KSHV LANA - Q76SB0; MHV-68 LANA - O41974; BRD4 - O60885. RCSB PDB Identifiers (previously published data): HPV-16 E2 CTD - 1BY9; EBV EBNA-1 - 1VHI; EBV EBNA-1 with DNA - 1B3T; BRD4 ET Domain: 2JNS.

## Supporting Information

Figure S1 **Extended sequence alignment of the KSHV LANA DNA Binding domain with chosen orthologs (related to** **Figure 1** **).** **A**: Islands of electron density at the dimerization interface of the kLANA CTD were interpreted as a water cluster in the monoclinic crystal form. **B**: Crystal structure of the dimeric mLANA CTD, front view. **C**: Residues at the sequence-specific DNA binding site of mLANA, bottom view. **D**: Top: Sequence alignment of the KSHV LANA C-terminal core domain with orthologs of three other γ2-herpesviruses, namely retroperitoneal fibromatosis herpesvirus (RFHVMn), rhesus rhadinovirus (RRV), and herpesvirus saimiri (HVS). Below: Structure-based extension of the alignment with murine herpesvirus 68 (MHV-68) LANA, Epstein-Barr virus (EBV) EBNA-1, and human papillomavirus 16 (HPV-16) E2. The dimeric structures were superimposed at conserved secondary structure elements. Cα distances to the corresponding residues of kLANA are shown as grey bars. Missing data or distances larger than 10 Å are indicated (*). Residues identical to kLANA are labeled with a dot below. **E**: Percentage of sequence identity for the given alignment. **F**: EMSA with LBS1+2 oligonucleotide and GST-LANA(934-1162) DNA binding deficient mutants. (wt+comp.) control with 10 fold excess of unlabeled probe. Right: Expression of the GST-LANA CTD proteins; Coomassie stained SDS PAGE gel. **G**: Transient replication assay with kLANA DNA binding deficient mutants and pTR1 vector in HeLa cells. Panel I: Southern blot of replicated DNA, remaining after digest with KpnI and DpnI. Panel II: Southern blot of input DNA linearized with KpnI; pBluescript (pBS) does not replicate and serves as internal control. Assay was performed in duplicates. (-) empty vector control. Panel III: LANA protein expression. Panel IV: Actin loading control. **H**: Oligomerization assay with kLANA DNA binding deficient mutants. Top left: Western blot detecting FL kLANA wt or mutants bound to GST-fused kLANA wt or mutant CTDs. Bottom left: pulled down GST-LANA CTD proteins in the same assay. (e.v.) empty vector, (-) GST without fusion protein, (MUT) mutant GST-LANA CTD always corresponding to the FL LANA mutant indicated above. Right: Expression of FL LANA proteins in eukaryotic cells (panel I) and the corresponding actin loading control (panel II).
(PDF)

Figure S2 **Details on the ET interacting site of KSHV LANA (related to** **Figure 4** **).** **A**: View at the cleft below the α3/β4 loop of kLANA. Islands of electron density were interpreted as chloride ions or a sulfate ion in two different crystal forms. Structural considerations strongly suggest that such ions would be replaced by the DNA phosphate backbone upon sequence-specific DNA binding. The 2F_o_-F_c_ maps are displayed at a contour level of 2.0 σ. **B**: kLANA wt or ‘ET binding site’ mutants were co-immunoprecipitated with GFP-tagged BRD2 full-length (FL), BRD2 C-terminal domain (CT; aa640-801), and BRD2 ET domain (ET; aa640-719). Panel I: Immunoblot of co-IP samples detecting LANA. Panel II: Blot of the same samples detecting GFP-BRD2 fragments. Panel III: Expression of LANA in all of the samples. Panel IV: Actin loading control. See also Figure S3C. **C**: Four double-point charge inversion kLANA CTD mutants were tested for their ability to induce specific chemical shift perturbations in the BRD4 ET domain. Left: Positions of the mutations on the kLANA CTD. Acidic residues are red and basic residues are blue. Right: Details of [^1^H,^15^N]-HSQC spectra of 0.15 mM ^15^N-BRD4(600-680) in 200 mM NaCl in the absence (black) and presence (orange) of 0.30 mM unlabeled kLANA(1013-1149) wild type or mutants. Chemical shift perturbations are indicated by arrows. **D**: Top: Energy plots of all 1000 models created in a local docking search (Rosetta Dock). The energy scores of the models are plotted against the deviation from the starting position. The plot shows two distinct energy minima. Below: Starting condition as well as the complex models at the two energy minima. Chemical shift perturbations are mapped in orange on the kLANA structure. On the ET domain, charged residues at or near the site of strongest chemical shift perturbations are shown in red (acidic) and blue (basic). The locations of three residues showing strong chemical shift perturbation upon kLANA binding are indicated. Also the region of strong chemical shift perturbation detectable only at 50 mM NaCl is indicated (*), compare Figure 3E. **E**: Oligomerization assay with kLANA ‘ET binding site’ mutants. Upper top: Western blot detecting FL kLANA wt or mutants bound to GST-fused kLANA wt or mutant CTDs. Lower top: Pulled down GST-LANA CTD proteins in the same assay. (e.v.) empty vector, (-) GST without fusion protein, (MUT) mutant GST-LANA CTD always corresponding to the FL LANA mutant indicated above. Bottom: Expression of FL LANA proteins in eukaryotic cells (panel I) and the corresponding actin loading control (panel II).
(PDF)

Figure S3 **Fine mapping of the LANA-interacting elements of BET proteins (related to** **Figure 5** **).** **A**: kLANA ‘basic top’ mutants were co-immunoprecipitated with GFP-BRD4 (HUNK; left) and GFP-BRD2 (right). Panels I for both BRD2 and BRD4 interaction assays represent immunoblots of co-IP samples detecting LANA. Panels II: Blot of the same samples with αGFP antibody detecting GFP-BET proteins. Panels III: Expression of LANA in all of the samples. Panels IV: Actin loading control; (*) nonspecific bands appearing with some αGFP antibody lots. **B**: Sequence alignment of BRD2 and BRD4 ET domains together with C-terminally flanking sequences. Similar fragments were previously positive in binding experiments with kLANA [26]. Acidic residues are red and basic residues are blue, serines are in boldface. **C**: kLANA was co-immunoprecipitated with GFP tagged BRD2 full length (FL), a C-terminal BRD2 fragment (CT), and deletion mutants thereof (a–g). Panel I: Immunoblot of co-IP samples with αLANA antibody showing the interaction. Panel II: Expression of the GFP tagged BRD2 proteins. Panel III: Expression of LANA. The analysis demonstrates that the globular ET domain is required and sufficient for specific binding to LANA (fragment ‘a’). The acidic serine-rich stretch increases binding affinity significantly (compare fragment CT with fragments ‘e’, ‘f’, ‘g’), but does not specifically bind to LANA when isolated from a functional ET domain (fragments ‘b’ and ‘c’). Fragment ‘d’ likely suffers from stability problems since helix α3 is truncated. **D**: Top: BRD4 ET domain fragments used in this study for NMR spectroscopy. Below: [^1^H,^15^N]-HSQC spectra of each 0.25 mM BRD4 fragments in the absence (black) and presence (orange) of 0.25 mM unlabeled kLANA CTD at 100 mM NaCl. BRD4(600-722) includes the acidic serine-rich stretch (top), whereas BRD4(608-680) does not (below). For the latter, moderate peak broadening and chemical shift perturbations are indicative of binding. However, when the acidic serine-stretch is included, massive peak broadening leads to loss of all resonances of the globular ET domain (top). Only resonances of flexible residues remain. This indicates formation of a large stable complex.
(PDF)

Figure S4 **Mapping of functional properties on the structure of the KSHV LANA CTD.** **A**: Model of two kLANA CTD dimers simultaneously bound to LBS1 and LBS2. The arrangement of the protein subunits is as found in the monoclinic crystal form. The DNA is bent by 100° to fit their curvature. **B**: Superposition of two chosen segments (in magenta and cyan, respectively) of the pentameric ring of kLANA CTD dimers as found in the monoclinic crystal form. Since the pentameric ring is not perfectly even, substantial variability in the relative dimer orientations in an angle perpendicular to the ring plane can be observed. It is thus conceivable that oligomeric assemblies other than rings can exist *in vivo*. **C**: First column: Surface electrostatic potential on the kLANA CTD in top view (top), bottom view (middle), and front view (bottom). Second column: Mean similarity score (BLOSUM62) in the structure-based alignment with LANA CTDs of RFHVMn, RRV, HVS, and MHV-68 on the surface of the KSHV LANA CTD (Figure S1D). Conserved residues cluster to the sequence-specific DNA binding site on the bottom of the dimer and to the ‘basic top’. Third column: Chemical shift perturbations upon interaction with the BRD4 ET domain on the structure of the kLANA CTD. Prolines and other unassigned residues are gray. Fourth column: Multiple alanine substitution mutations leading to loss of C-terminal chromosome association [24] on the structure of the kLANA CTD. Alanines and other residues which had not been mutated in the study are gray. Fifth column: Multiple alanine substitution mutations are color-coded according to their behavior in EMSA with an LBS1 probe [24]. Mutations leading to decreased binding efficiency are in magenta and mutations leading to increased binding are in green. Alanines and other residues, which had not been mutated in the study, are gray. Fifth column, bottom: Model of the kLANA CTD bound in a sequence specific manner to a single LBS; front view.
(PDF)

Table S1 **Data processing and refinement statistics (related to** **Figures 1** **, S1).** Table lists parameters for the four different crystal structures presented in this work.
(DOCX)

Table S2 **Summary of functional assays with KSHV LANA mutants in this study.** Table summarizes results of all functional assays (including: EMSA with LBS1+2, GST pull down based oligomerization assay, co-IP testing binding to Brd2 and 4, transient replication assay and speckle formation assay) performed with mutants targeting different LANA surfaces.
(DOCX)
