# Supplementary figures and images for: A Structural Basis for BRD2/4-Mediated Host Chromatin Interaction and Oligomer Assembly of Kaposi Sarcoma-Associated Herpesvirus and Murine Gammaherpesvirus LANA Proteins
Source: PLoS Pathog. 2013 Oct 17;9(10):e1003640. doi: 10.1371/journal.ppat.1003640 (PMC3798688; doi:10.1371/journal.ppat.1003640)

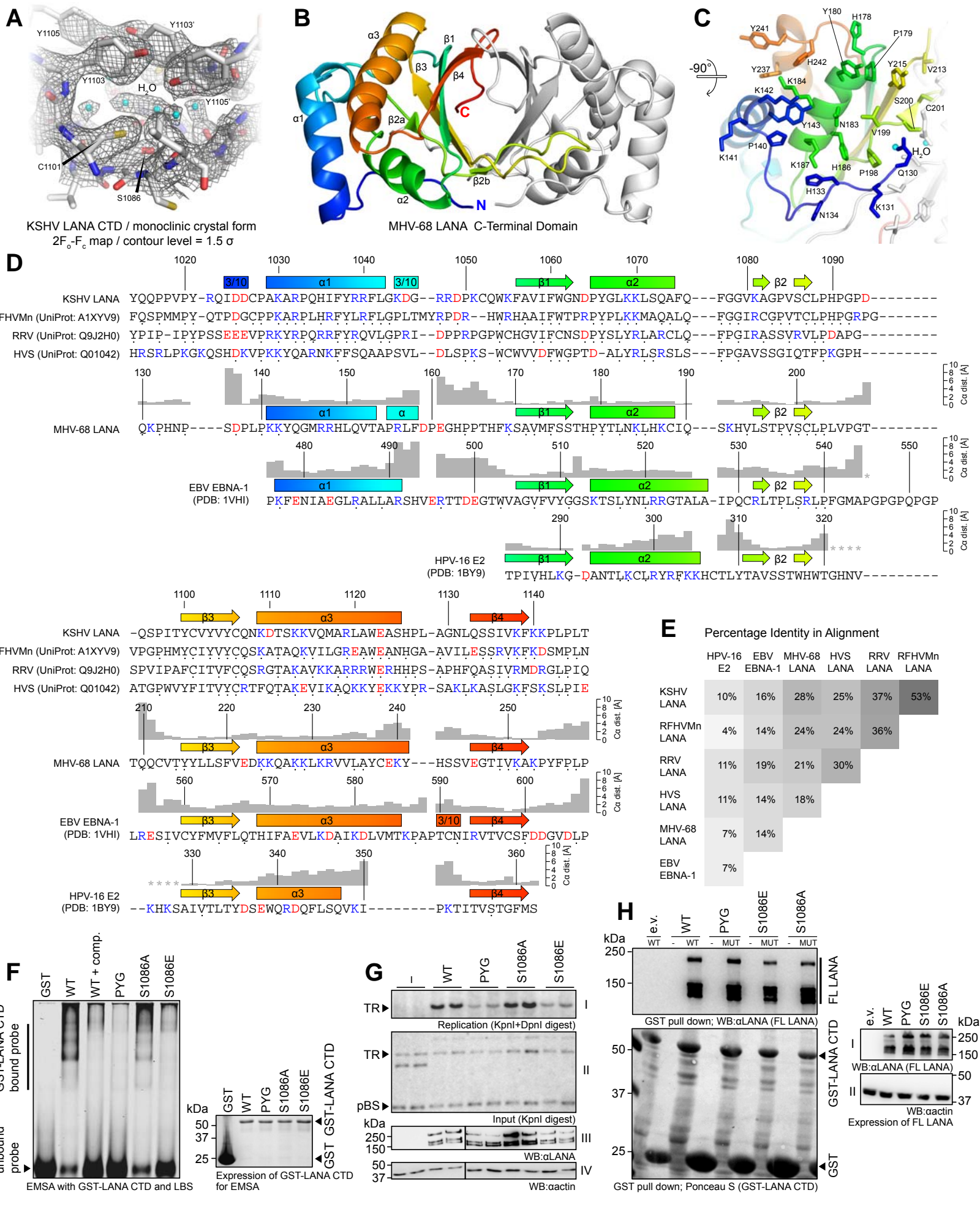

Supplement: Figure S1 — Extended sequence alignment of the KSHV LANA DNA Binding domain with chosen orthologs (related to Figure 1 ). A: Islands of electron density at the dimerization interface of the kLANA CTD were interpreted as a water cluster in the monoclinic crystal form. B: Crystal structure of the dimeric mLANA CTD, front view. C: Residues at the sequence-specific DNA binding site of mLANA, bottom view. D: Top: Sequence alignment of the KSHV LANA C-terminal core domain with orthologs of three other γ2-herpesviruses, namely retroperitoneal fibromatosis herpesvirus (RFHVMn), rhesus rhadinovirus (RRV), and herpesvirus saimiri (HVS). Below: Structure-based extension of the alignment with murine herpesvirus 68 (MHV-68) LANA, Epstein-Barr virus (EBV) EBNA-1, and human papillomavirus 16 (HPV-16) E2. The dimeric structures were superimposed at conserved secondary structure elements. Cα distances to the corresponding residues of kLANA are shown as grey bars. Missing data or distances larger than 10 Å are indicated (*). Residues identical to kLANA are labeled with a dot below. E: Percentage of sequence identity for the given alignment. F: EMSA with LBS1+2 oligonucleotide and GST-LANA(934-1162) DNA binding deficient mutants. (wt+comp.) control with 10 fold excess of unlabeled probe. Right: Expression of the GST-LANA CTD proteins; Coomassie stained SDS PAGE gel. G: Transient replication assay with kLANA DNA binding deficient mutants and pTR1 vector in HeLa cells. Panel I: Southern blot of replicated DNA, remaining after digest with KpnI and DpnI. Panel II: Southern blot of input DNA linearized with KpnI; pBluescript (pBS) does not replicate and serves as internal control. Assay was performed in duplicates. (-) empty vector control. Panel III: LANA protein expression. Panel IV: Actin loading control. H: Oligomerization assay with kLANA DNA binding deficient mutants. Top left: Western blot detecting FL kLANA wt or mutants bound to GST-fused kLANA wt or mutant CTDs. Bottom left: pulled down GST- [file ppat.1003640.s001.pdf]

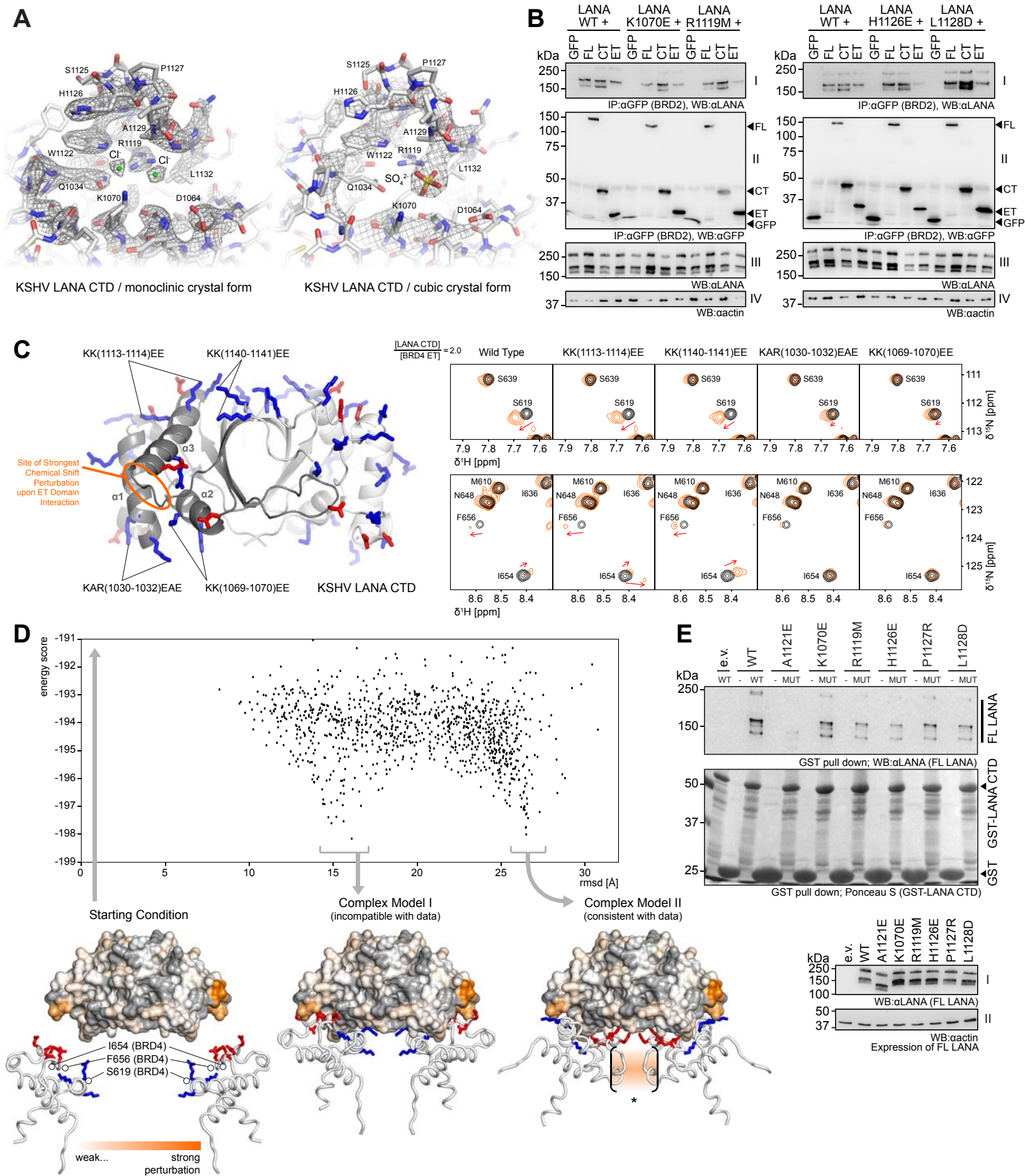

Supplement: Figure S2 — Details on the ET interacting site of KSHV LANA (related to Figure 4 ). A: View at the cleft below the α3/β4 loop of kLANA. Islands of electron density were interpreted as chloride ions or a sulfate ion in two different crystal forms. Structural considerations strongly suggest that such ions would be replaced by the DNA phosphate backbone upon sequence-specific DNA binding. The 2Fo-Fc maps are displayed at a contour level of 2.0 σ. B: kLANA wt or ‘ET binding site’ mutants were co-immunoprecipitated with GFP-tagged BRD2 full-length (FL), BRD2 C-terminal domain (CT; aa640-801), and BRD2 ET domain (ET; aa640-719). Panel I: Immunoblot of co-IP samples detecting LANA. Panel II: Blot of the same samples detecting GFP-BRD2 fragments. Panel III: Expression of LANA in all of the samples. Panel IV: Actin loading control. See also Figure S3C. C: Four double-point charge inversion kLANA CTD mutants were tested for their ability to induce specific chemical shift perturbations in the BRD4 ET domain. Left: Positions of the mutations on the kLANA CTD. Acidic residues are red and basic residues are blue. Right: Details of [1H,15N]-HSQC spectra of 0.15 mM 15N-BRD4(600-680) in 200 mM NaCl in the absence (black) and presence (orange) of 0.30 mM unlabeled kLANA(1013-1149) wild type or mutants. Chemical shift perturbations are indicated by arrows. D: Top: Energy plots of all 1000 models created in a local docking search (Rosetta Dock). The energy scores of the models are plotted against the deviation from the starting position. The plot shows two distinct energy minima. Below: Starting condition as well as the complex models at the two energy minima. Chemical shift perturbations are mapped in orange on the kLANA structure. On the ET domain, charged residues at or near the site of strongest chemical shift perturbations are shown in red (acidic) and blue (basic). The locations of three residues showing strong chemical shift perturbation upon kLANA binding are indicated. Also the region of [file ppat.1003640.s002.pdf]

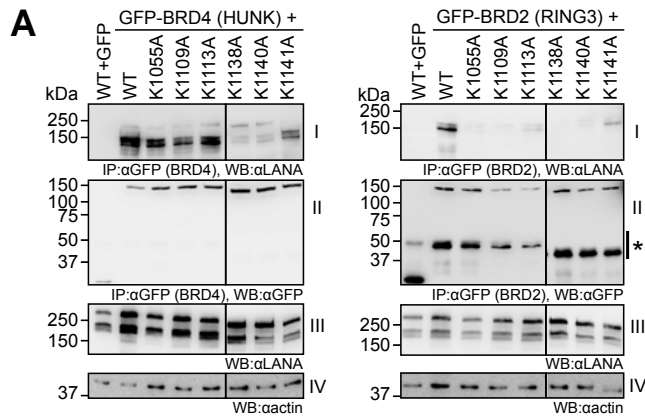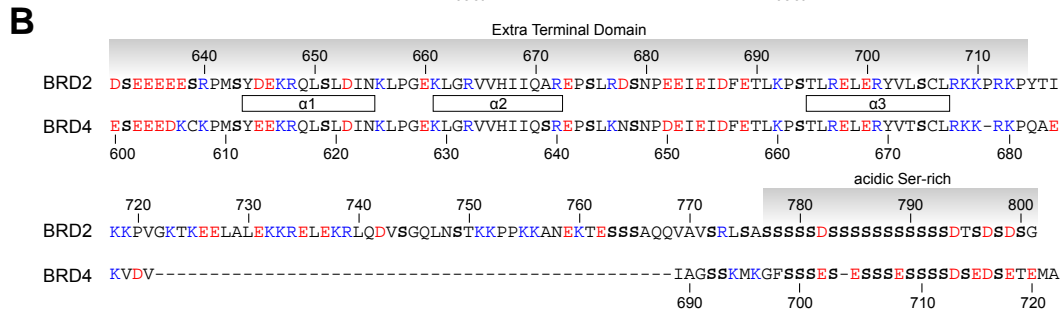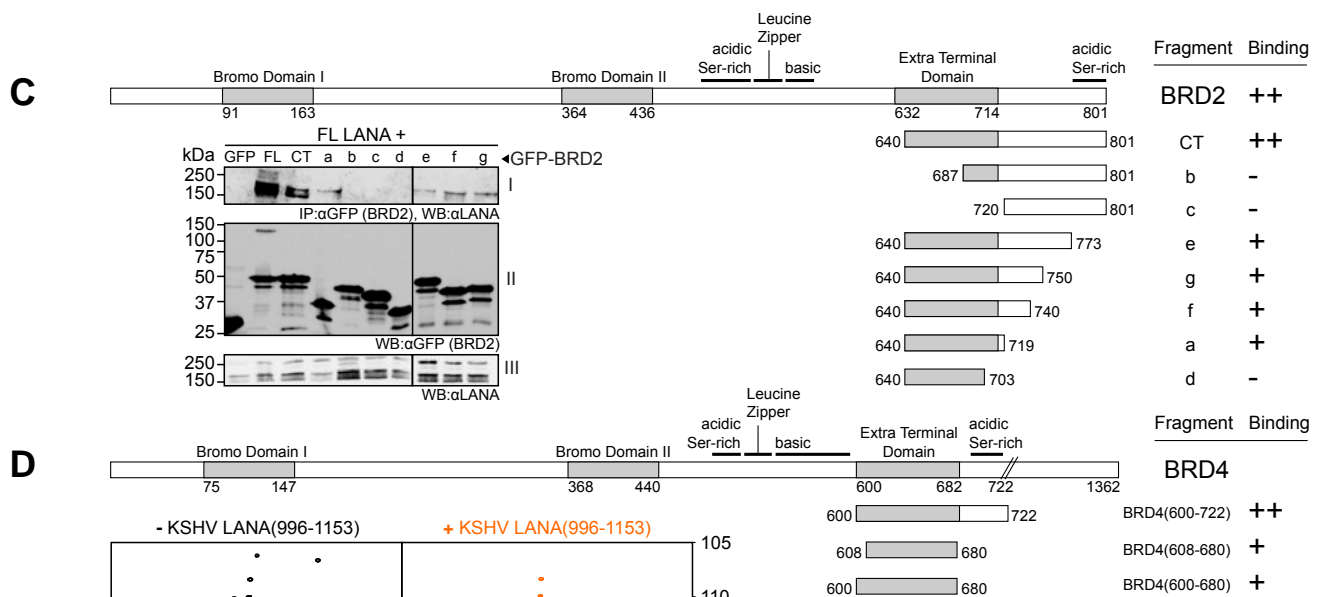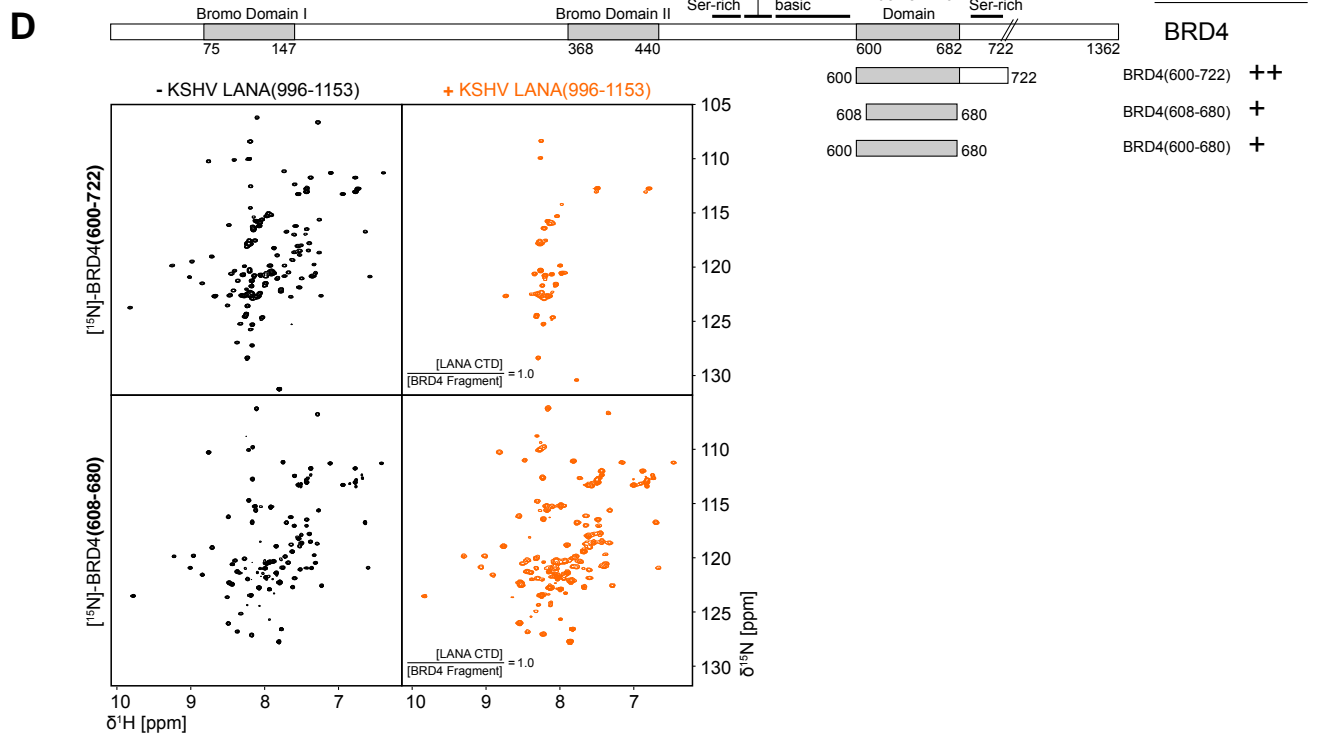

Supplement: Figure S3 — Fine mapping of the LANA-interacting elements of BET proteins (related to Figure 5 ). A: kLANA ‘basic top’ mutants were co-immunoprecipitated with GFP-BRD4 (HUNK; left) and GFP-BRD2 (right). Panels I for both BRD2 and BRD4 interaction assays represent immunoblots of co-IP samples detecting LANA. Panels II: Blot of the same samples with αGFP antibody detecting GFP-BET proteins. Panels III: Expression of LANA in all of the samples. Panels IV: Actin loading control; (*) nonspecific bands appearing with some αGFP antibody lots. B: Sequence alignment of BRD2 and BRD4 ET domains together with C-terminally flanking sequences. Similar fragments were previously positive in binding experiments with kLANA [26]. Acidic residues are red and basic residues are blue, serines are in boldface. C: kLANA was co-immunoprecipitated with GFP tagged BRD2 full length (FL), a C-terminal BRD2 fragment (CT), and deletion mutants thereof (a–g). Panel I: Immunoblot of co-IP samples with αLANA antibody showing the interaction. Panel II: Expression of the GFP tagged BRD2 proteins. Panel III: Expression of LANA. The analysis demonstrates that the globular ET domain is required and sufficient for specific binding to LANA (fragment ‘a’). The acidic serine-rich stretch increases binding affinity significantly (compare fragment CT with fragments ‘e’, ‘f’, ‘g’), but does not specifically bind to LANA when isolated from a functional ET domain (fragments ‘b’ and ‘c’). Fragment ‘d’ likely suffers from stability problems since helix α3 is truncated. D: Top: BRD4 ET domain fragments used in this study for NMR spectroscopy. Below: [1H,15N]-HSQC spectra of each 0.25 mM BRD4 fragments in the absence (black) and presence (orange) of 0.25 mM unlabeled kLANA CTD at 100 mM NaCl. BRD4(600-722) includes the acidic serine-rich stretch (top), whereas BRD4(608-680) does not (below). For the latter, moderate peak broadening and chemical shift perturbations are indicative of binding. However, when the acidic serine-stret [file ppat.1003640.s003.pdf]

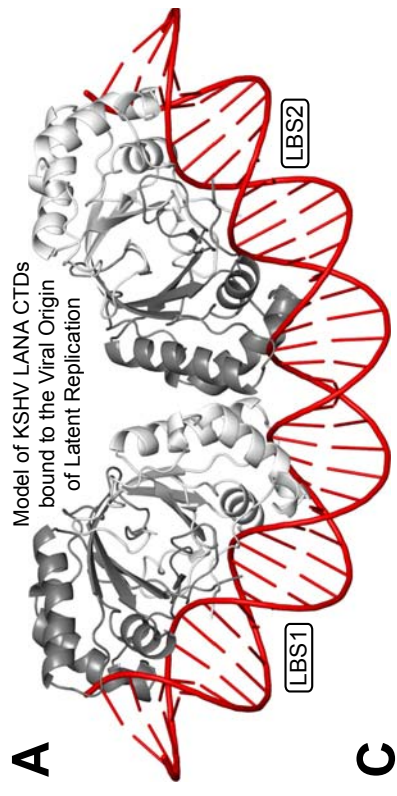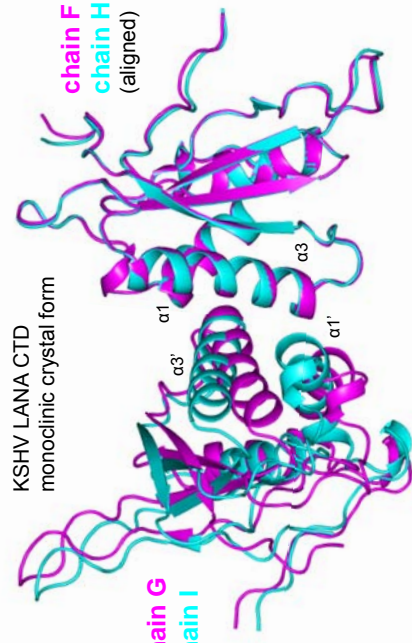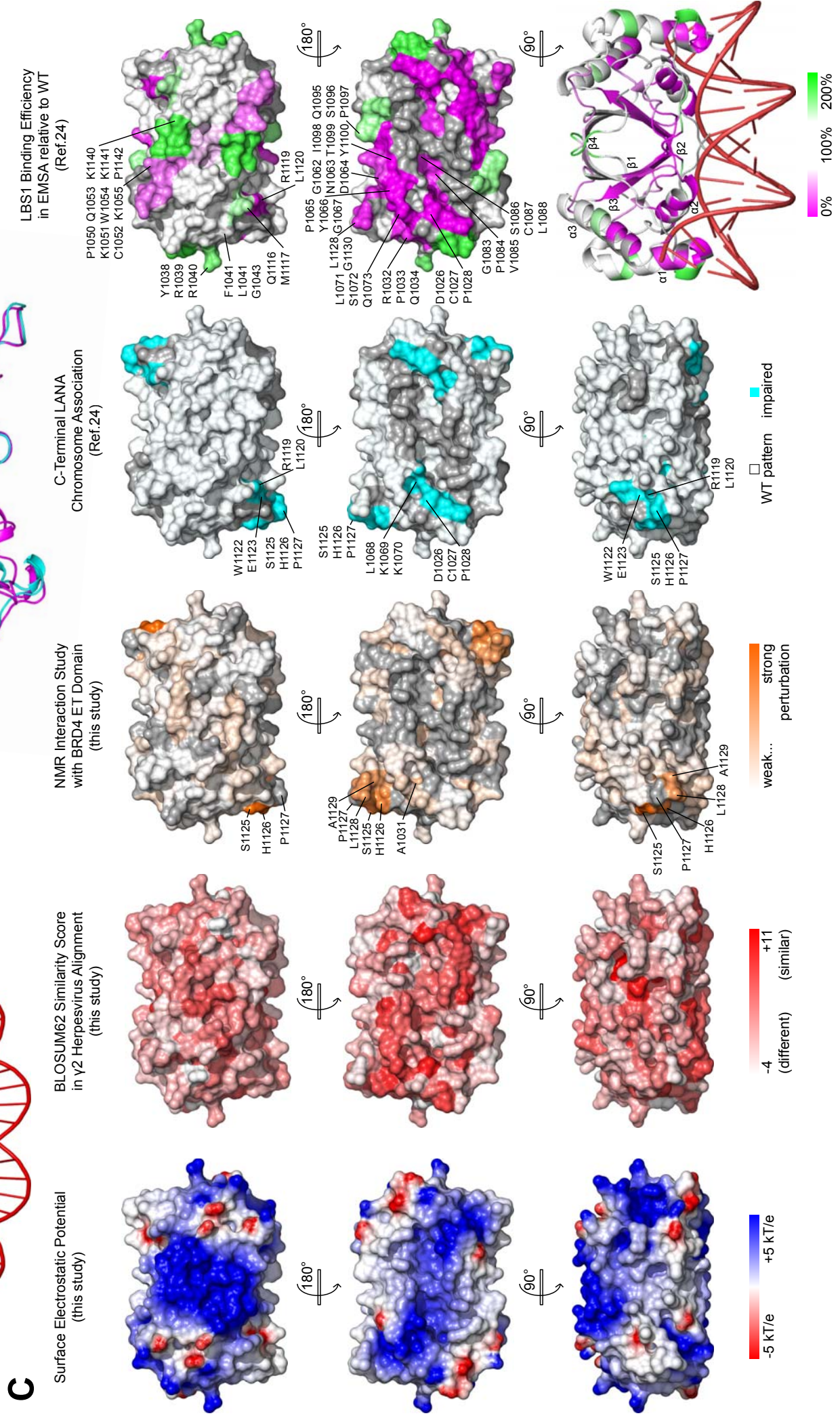

Supplement: Figure S4 — Mapping of functional properties on the structure of the KSHV LANA CTD. A: Model of two kLANA CTD dimers simultaneously bound to LBS1 and LBS2. The arrangement of the protein subunits is as found in the monoclinic crystal form. The DNA is bent by 100° to fit their curvature. B: Superposition of two chosen segments (in magenta and cyan, respectively) of the pentameric ring of kLANA CTD dimers as found in the monoclinic crystal form. Since the pentameric ring is not perfectly even, substantial variability in the relative dimer orientations in an angle perpendicular to the ring plane can be observed. It is thus conceivable that oligomeric assemblies other than rings can exist in vivo. C: First column: Surface electrostatic potential on the kLANA CTD in top view (top), bottom view (middle), and front view (bottom). Second column: Mean similarity score (BLOSUM62) in the structure-based alignment with LANA CTDs of RFHVMn, RRV, HVS, and MHV-68 on the surface of the KSHV LANA CTD (Figure S1D). Conserved residues cluster to the sequence-specific DNA binding site on the bottom of the dimer and to the ‘basic top’. Third column: Chemical shift perturbations upon interaction with the BRD4 ET domain on the structure of the kLANA CTD. Prolines and other unassigned residues are gray. Fourth column: Multiple alanine substitution mutations leading to loss of C-terminal chromosome association [24] on the structure of the kLANA CTD. Alanines and other residues which had not been mutated in the study are gray. Fifth column: Multiple alanine substitution mutations are color-coded according to their behavior in EMSA with an LBS1 probe [24]. Mutations leading to decreased binding efficiency are in magenta and mutations leading to increased binding are in green. Alanines and other residues, which had not been mutated in the study, are gray. Fifth column, bottom: Model of the kLANA CTD bound in a sequence specific manner to a single LBS; front view. (PDF) [file ppat.1003640.s004.pdf]
