# Supplementary material for: A Structural Basis for BRD2/4-Mediated Host Chromatin Interaction and Oligomer Assembly of Kaposi Sarcoma-Associated Herpesvirus and Murine Gammaherpesvirus LANA Proteins
Source: PLoS Pathog. 2013 Oct 17;9(10):e1003640. doi: 10.1371/journal.ppat.1003640 (PMC3798688; doi:10.1371/journal.ppat.1003640)
Supplement: Table S1 — Data processing and refinement statistics (related to Figures 1 , S1). Table lists parameters for the four different crystal structures presented in this work. (DOCX) [file ppat.1003640.s005.docx]

| **Parameter** | **KSHV LANA(1013-1149)** | | | | **KSHV LANA(996-1153)** | **MHV-68 LANA(124-260)** |
| --- | --- | --- | --- | --- | --- | --- |
| Crystal form | monoclinic | | | orthorhombic | cubic | triclinic |
| Data set | anomalous | native | | native | native | native |
| Space group | P2_1_ | | | P2_1_2_1_2_1_ | P432 | P1 |
| Unit cell axes dimensions: *a, b, c* (Å) | 51.7, 175.9, 97.6 | | | 76.8, 100.3, 214.6 | 107.4, 107.4, 107.4 | 36.0, 53.4, 70.9 |
| Unit cell inclination angles: α, β, γ (°) | 90.0, 95.5, 90.0 | | | 90.0, 90.0, 90.0 | 90.0, 90.0, 90.0 | 106.0, 93.5, 109.8 |
| X-ray source | BESSY BL 14.2 | | | Rigaku MicroMax-007 HF | BESSY BL 14.2 | BESSY BL 14.2 |
| Wavelength (Å) | 0.91841 | | 1.07208 | 1.54181 | 0.91841 | 0.91841 |
| Resolution range (Å) | 20.00-3.00 (3.08-3.00)^a^ | | 20.00-2.45 (2.53-2.45)^b^ | 50.00-3.20 (3.39-3.20) | 35.00-3.91 (4.01-3.91) | 50.00-2.14 (2.27-2.14) |
| R_merge_^c^ | 0.06 (0.57) | | 0.05 (0.48) | 0.19 (0.61) | 0.11 (0.73) | 0.10 (0.38) |
| Mean I/σI | 25.37 (3.64) | | 16.24 (2.60) | 7.67 (2.01) | 21.73 (3.92) | 7.40 (2.34) |
| No. of unique reflections | 67,866 | | 54,311 | 27,442 | 2,178 | 24,125 |
| Completeness | 0.988 (0.999) | | 0.857 (0.210) | 0. 975 (0.951) | 0. 997 (1.000) | 0. 928 (0.808) |
| Multiplicity | 7.4 (7.5) | | 3.8 (3.6) | 3.5 (3.4) | 10.8 (11.1) | 2.0 (2.0) |
| Content of au | 10 monomers | | | 10 monomers | 1 monomer | 4 monomers |
| R_work_^d^ | 0.2207 | | | 0.2200 | 0.2595 | 0.2024 |
| R_free_^e^ | 0.2522 | | | 0.2650 | 0.3166 | 0.2456 |
| r.m.s.d. bond lengths (Å) | 0.003 | | | 0.003 | 0.003 | 0.005 |
| r.m.s.d. angles (°) | 0.645 | | | 0.734 | 0.746 | 1.009 |
| Ramachandran plot - favored (%) | 99.2 | | | 98.8 | 97.4 | 97.2 |
| Ramachandran plot - allowed (%) | 0.8 | | | 1.2 | 2.6 | 2.8 |
| Ramachandran plot - disallowed (%) | 0.0 | | | 0.0 | 0.0 | 0.0 |

**Table S1. Data Processing and Refinement Statistics (related to Figures 1, S1)**

Values in parenthesis account for the shell with the highest resolution.

a) The anomalous signal was good to 3.5 Å resolution.

b) The structure is reported as of 2.6 Å resolution. Data of higher resolution were included in the refinement but show completeness below 90%.

c) R_merge_= Σ|I_o_ - Σ⟨I⟩| / Σ I_o_

d) R_work_ = (Σ ||F_o_|-|F_c_|| / Σ |F_o_|)

e) R_free_ is computed as R_work_ but using 5% randomly assigned reflections excluded from refinement.
