# Supplementary material for: A Structural Basis for BRD2/4-Mediated Host Chromatin Interaction and Oligomer Assembly of Kaposi Sarcoma-Associated Herpesvirus and Murine Gammaherpesvirus LANA Proteins
Source: PLoS Pathog. 2013 Oct 17;9(10):e1003640. doi: 10.1371/journal.ppat.1003640 (PMC3798688; doi:10.1371/journal.ppat.1003640)
Supplement: Table S2 — Summary of functional assays with KSHV LANA mutants in this study. Table summarizes results of all functional assays (including: EMSA with LBS1+2, GST pull down based oligomerization assay, co-IP testing binding to Brd2 and 4, transient replication assay and speckle formation assay) performed with mutants targeting different LANA surfaces. (DOCX) [file ppat.1003640.s006.docx]

**Table S2. Summary of Functional Assays with KSHV LANA Mutants in This Study**

| **KSHV LANA protein category** | **KSHV LANA protein** | **LBS DNA binding** | **Oligomerization (pull down)** | **Co-IP BRD4** | **Co-IP BRD2** | **Transient replication** | **Speckle formation** |
| --- | --- | --- | --- | --- | --- | --- | --- |
| **Control** | wt | + | + | + | + | + | + |
| **Specific DNA binding site** | PYG1065-0167AAA | - | + | ++ | ++ | - | - |
|  | S1086E | - | + | +/- | +/- | - | - |
|  | S1086A | + | + | + | + | + | +/- |
| **Oligomerization interface** | M1117D | + | +/- | + | + | - | n.d. |
|  | A1121E | + | - | + | + | - | - |
| **‘ET binding site’** | R1119M | + | + | - | - | - | - |
|  | P1127R | + | + | + | + | + | + |
|  | K1070E | +/- | + | + | + | - | - |
|  | L1128D | + | + | + | + | - | (+) |
|  | H1126E | + | + | ++ | ++ | - | - |
| **‘Basic top’** | K1109A/K1138A | + | - | - | - | +/- | - |
|  | K1109A | + | +/- | - | - | + | + |
|  | K1138A | + | - | - | - | + | + |
|  | K1113A | + | +/- | + | - | + | + |
|  | K1140A | + | +/- | - | - | + | n.d. |
|  | K1141A | + | + | + | +/- | + | n.d. |
|  | K1055A | n.d. | ++ | + | - | + | + |

n.d.: not determined

+/-, (+): intermediate phenotypes
